# Supplementary material for: The impact of university students’ computational thinking on AI literacy: A longitudinal study based on SEM-PLS
Source: PLoS One. 2026 May 27;21(5):e0350124. doi: 10.1371/journal.pone.0350124 (PMC13215501; doi:10.1371/journal.pone.0350124)
Supplement: S1 Data — (PDF) [file pone.0350124.s001.pdf]

T0-Index	Group	T0-Gender	T0-Grade	T0-Subject	CT1	CT2	CT3	CT4
1	1	2	4	2	4	2	4	4
2	1	2	4	1	3	2	3	3
3	1	2	4	1	4	5	5	3
4	1	2	4	2	3	2	2	2
5	1	2	3	1	2	3	4	3
6	1	2	3	2	4	5	5	5
7	1	2	3	1	4	5	4	4
8	1	2	2	1	2	2	4	3
9	1	2	3	2	2	3	3	3
10	1	2	3	1	4	5	5	3
11	1	2	3	1	3	4	3	2
12	1	2	4	1	3	5	5	4
13	1	2	2	1	3	3	4	3
14	1	1	3	2	3	3	2	3
15	1	2	4	1	2	3	3	2
16	1	2	4	2	4	3	3	3
17	1	2	3	2	1	1	1	1
18	1	2	3	1	2	1	2	2
19	1	2	3	1	3	2	4	3
20	1	2	3	2	4	4	4	4
21	1	1	3	1	2	5	3	3
22	1	2	3	2	3	3	3	2
23	1	2	4	1	3	4	4	2
24	1	1	2	2	3	3	3	4
25	1	2	4	1	2	2	5	2
26	1	2	4	1	4	4	4	3
27	1	2	3	2	2	4	4	3
28	1	1	4	2	3	2	4	4
29	1	2	2	2	3	2	3	3
30	1	1	1	1	2	2	3	3
31	1	2	1	2	4	4	4	4
32	1	1	1	2	5	5	5	5
33	1	2	1	1	3	5	3	3
34	1	1	3	2	5	4	4	4
35	1	2	1	2	3	3	3	2
36	1	1	1	1	5	5	5	5
37	1	2	3	1	1	2	2	2
38	1	1	3	2	2	2	3	3
39	1	1	3	2	3	3	3	4
40	1	2	4	1	3	2	4	2
41	1	2	2	2	2	2	3	3
42	1	1	4	1	2	2	3	3
43	1	1	2	2	4	3	3	4
44	1	2	2	1	3	3	3	4
45	1	2	3	1	3	2	2	2
46	1	1	3	2	5	5	5	5
47	1	1	4	2	3	3	3	3
48	1	2	4	1	3	3	3	3
49	1	2	3	1	5	5	3	3
50	1	2	1	2	3	3	3	3
51	1	1	1	2	4	5	4	4
52	1	1	3	2	3	4	4	3
53	1	2	3	1	3	3	3	3
54	1	1	1	2	5	5	5	5
55	1	2	3	2	2	2	3	4

56	1	2	1	2	3	3	3	3
57	1	2	1	2	4	4	4	4
58	1	2	3	2	3	4	3	3
59	1	1	3	2	4	4	4	4
60	1	2	1	2	4	5	4	3
61	1	2	1	1	4	5	5	4
62	1	2	1	1	3	2	3	2
63	1	2	3	2	2	3	3	2
64	1	1	1	2	5	5	5	5
65	1	1	3	2	4	4	3	4
66	1	2	3	2	3	4	4	3
67	1	2	1	2	4	4	4	3
68	1	1	1	2	3	4	4	4
69	1	2	3	2	2	3	3	3
70	1	2	1	2	4	4	4	4
71	1	2	2	1	1	3	2	2
72	1	1	2	2	3	3	4	3
73	1	2	2	1	2	4	3	5
74	1	2	2	1	2	3	2	2
75	1	1	2	1	3	2	3	3
76	1	1	2	2	2	2	2	3
77	1	2	3	2	4	4	4	4
78	1	1	3	2	3	4	3	3
79	1	1	3	2	4	4	4	4
80	1	1	3	2	1	3	3	3
81	1	1	3	2	2	3	3	3
82	1	1	3	1	4	4	4	4
83	1	1	3	2	4	4	4	4
84	1	1	3	2	3	3	3	3
85	1	1	3	1	4	4	3	4
86	1	2	3	1	2	4	4	4
87	1	1	3	2	3	3	3	3
88	1	1	3	2	2	3	3	3
89	1	1	3	1	2	2	2	2
90	1	2	1	2	2	3	4	3
91	1	1	3	2	4	4	4	4
92	1	1	2	2	3	3	3	3
93	1	1	3	2	5	5	4	4
94	1	1	3	1	4	5	3	3
95	1	2	2	2	3	3	3	4
96	1	2	3	2	3	3	4	3
97	1	2	3	1	1	1	1	1
98	1	2	2	1	3	2	4	2
99	1	1	3	2	4	4	4	4
100	1	1	2	2	3	4	3	3
101	1	2	1	2	3	3	3	3
102	1	2	3	2	3	3	5	3
103	1	2	3	2	3	5	4	3
104	1	1	3	2	3	4	2	5
105	1	2	1	2	3	5	5	2
106	1	1	3	2	4	4	4	4
107	1	2	1	1	4	4	4	4
108	1	1	3	2	2	4	3	3
109	1	2	3	2	3	4	4	4
110	1	1	3	2	4	5	4	4
111	1	1	1	2	2	3	3	3

112	1	1	3	2	3	4	5	3
113	1	2	1	1	3	4	3	3
114	1	1	1	2	3	3	3	3
115	1	2	1	2	3	4	4	3
116	1	1	3	2	4	4	5	3
117	1	1	3	1	3	4	4	4
118	1	1	1	2	5	5	5	5
119	1	2	3	2	2	4	5	3
120	1	2	1	2	3	4	3	3
121	1	2	4	2	3	4	4	4
122	1	1	4	2	3	3	4	4
123	1	1	3	2	2	3	4	3
124	1	2	1	2	3	4	4	3
125	1	2	1	2	4	4	4	4
126	1	2	3	2	3	3	2	2
127	1	2	3	2	3	5	5	4
128	1	1	1	2	2	3	3	3
129	1	2	3	1	4	4	4	4
130	1	1	1	1	2	3	3	3
131	1	1	3	2	4	4	4	4
132	1	1	1	2	4	3	4	3
133	1	1	3	2	3	3	4	4
134	1	1	1	2	2	3	3	2
135	1	1	3	2	3	5	5	5
136	1	1	3	2	4	3	4	3
137	1	1	3	1	5	5	5	5
138	1	2	1	1	3	3	3	3
139	1	2	1	1	3	3	4	3
140	1	2	3	1	3	4	4	4
141	1	2	3	1	2	4	3	2
142	1	1	1	2	3	3	4	3
143	1	1	3	1	2	2	2	2
144	1	2	1	1	3	3	3	3
145	1	2	1	1	4	5	4	4
146	1	2	3	2	3	4	3	3
147	1	2	1	2	3	4	3	3
148	1	1	3	1	4	4	5	4
149	1	1	3	1	4	4	4	4
150	1	1	1	2	3	4	5	3
151	1	1	1	1	3	4	3	3
152	1	2	3	1	3	3	3	3
153	1	2	3	2	3	3	5	3
154	1	1	1	2	4	5	5	3
155	1	2	3	2	3	5	5	4
156	1	1	3	2	3	5	3	3
157	1	1	1	2	3	5	3	4
158	1	2	3	2	3	3	3	3
159	1	2	3	2	2	3	3	3
160	1	1	3	2	3	4	3	4
161	1	2	1	2	3	5	4	3
162	1	2	1	2	4	4	4	4
163	1	1	3	2	3	5	4	3
164	1	2	3	2	3	4	3	4
165	1	2	1	2	2	2	3	2
166	1	2	3	2	2	4	4	2
167	1	2	3	2	3	4	3	4

168	1	2	1	2	3	3	4	3
169	1	2	1	2	4	4	4	4
170	1	2	1	2	2	3	4	3
171	1	1	1	2	3	4	4	3
172	1	1	2	2	3	3	3	3
173	1	2	3	1	3	3	5	3
174	1	1	3	2	2	1	2	1
175	1	2	2	2	2	3	4	4
176	1	2	2	2	3	3	3	3
177	1	2	2	2	2	3	4	2
178	1	2	1	2	2	2	2	3
179	1	1	2	2	2	2	3	3
180	1	2	1	2	4	4	4	4
181	1	2	2	2	4	4	4	3
182	1	1	2	1	3	3	3	3
183	1	2	2	2	3	3	4	4
184	1	2	2	2	3	3	3	3
185	1	2	2	1	3	3	4	3
186	1	2	2	1	4	4	4	3
187	1	1	2	2	3	3	3	3
188	1	2	2	2	2	4	5	4
189	1	2	2	1	2	5	3	2
190	1	2	2	2	3	3	3	3
191	1	2	2	2	2	1	3	2
192	1	2	2	2	3	4	3	3
193	1	2	2	1	2	2	3	2
194	1	2	2	2	3	3	3	3
195	1	1	2	2	4	4	4	4
196	1	2	2	1	2	4	4	3
197	1	2	2	2	4	3	3	3
198	1	2	2	1	2	3	3	2
199	1	1	2	2	3	3	4	4
200	1	2	2	2	3	3	4	3
201	1	2	2	2	3	2	5	3
202	1	2	2	2	3	3	4	3
203	1	1	3	2	3	3	3	3
204	1	2	2	2	3	3	3	3
205	1	2	2	1	2	2	3	2
206	1	2	1	2	4	3	4	3
207	1	2	2	1	3	3	4	2
208	1	2	2	2	3	5	4	4
209	1	2	2	2	2	3	3	3
210	1	2	2	2	3	3	3	3
211	1	1	2	1	3	3	3	3
212	1	1	2	2	4	4	5	5
213	1	2	2	2	4	4	4	4
214	1	2	2	2	3	4	3	3
215	1	2	2	1	2	2	3	3
216	1	2	2	1	3	4	3	4
217	2	2	2	1	3	2	3	4
218	2	1	1	1	4	4	4	4
219	2	1	2	2	5	5	4	4
220	2	1	2	1	3	3	3	3
221	2	1	2	1	3	3	3	3
222	2	1	2	1	3	2	3	2
223	2	1	2	1	4	4	4	5

224	2	2	2	1	2	3	3	2
225	2	1	2	2	3	3	3	3
226	2	1	2	2	4	4	2	3
227	2	1	1	2	4	3	3	3
228	2	1	2	1	3	4	2	3
229	2	1	2	2	2	2	2	3
230	2	2	2	1	2	3	3	3
231	2	2	2	2	4	3	3	4
232	2	2	2	1	2	3	3	3
233	2	1	2	2	4	4	4	4
234	2	2	2	2	3	3	3	3
235	2	2	2	1	3	2	2	3
236	2	2	2	1	5	5	5	5
237	2	1	2	1	4	4	4	4
238	2	1	2	1	3	4	4	4
239	2	2	2	2	3	3	3	3
240	2	2	2	2	4	3	5	2
241	2	2	2	2	2	3	4	4
242	2	2	2	1	3	3	3	3
243	2	1	2	1	4	3	3	4
244	2	2	2	1	3	3	3	3
245	2	2	2	2	3	3	2	3
246	2	1	2	2	3	3	3	3
247	2	1	1	1	4	5	5	5
248	2	1	4	1	5	4	2	3
249	2	2	2	1	3	3	2	3
250	2	2	2	1	3	3	3	2
251	2	2	2	1	5	5	5	5
252	2	2	2	1	4	4	4	4
253	2	1	2	1	3	3	3	3
254	2	1	2	1	3	3	3	3
255	2	2	2	1	4	4	4	4
256	2	2	2	2	3	3	3	3
257	2	1	2	1	3	3	3	3
258	2	2	2	1	4	4	4	4
259	2	2	2	1	4	4	3	4
260	2	1	2	2	3	3	3	3
261	2	1	2	1	3	4	4	3
262	2	1	2	1	5	5	5	5
263	2	1	2	1	1	5	1	5
264	2	1	2	1	3	4	4	4
265	2	1	2	1	4	4	5	4
266	2	1	2	2	4	4	4	3
267	2	2	2	2	3	3	2	3
268	2	2	2	1	4	3	4	3
269	2	2	2	2	3	3	3	3
270	2	2	2	1	4	4	4	4
271	2	2	2	1	2	4	3	3
272	2	1	2	2	3	5	3	3
273	2	2	2	2	4	4	4	4
274	2	2	2	2	1	3	3	3
275	2	2	2	1	4	3	3	4
276	2	1	2	2	3	3	3	3
277	2	2	2	2	2	4	3	3
278	2	2	2	2	4	3	3	3
279	2	2	2	1	3	3	3	3

280	2	2	3	2	4	4	3	3
281	2	2	3	2	3	3	3	3
282	2	2	3	2	3	2	3	3
283	2	1	3	2	2	4	2	3
284	2	1	3	2	3	3	2	3
285	2	1	3	2	3	2	3	3
286	2	1	3	2	4	4	2	3
287	2	2	3	2	3	3	4	2
288	2	2	3	2	2	3	2	3
289	2	2	3	2	4	4	5	3
290	2	1	3	2	2	4	2	3
291	2	1	3	2	4	4	4	3
292	2	1	3	2	3	3	2	2
293	2	2	3	2	3	5	5	5
294	2	2	3	2	4	4	4	4
295	2	2	3	2	3	4	3	3
296	2	2	3	2	4	4	3	3
297	2	1	3	2	3	3	3	3
298	2	2	3	2	2	4	4	3
299	2	2	3	2	3	4	3	4
300	2	2	3	2	4	3	3	4
301	2	2	3	2	2	3	3	3

CT5	CT6	AIL1	AIL2	AIL3	AIL4	AIL5	AIL6	AIL7
4	4	3	4	3	4	2	3	4
4	3	3	3	3	2	2	3	2
4	4	4	5	5	4	4	3	5
2	2	1	3	2	2	2	2	3
3	3	2	3	3	2	2	2	2
4	4	4	4	4	3	3	4	3
4	4	4	4	4	4	4	4	3
3	3	3	3	3	3	3	3	2
4	4	4	4	4	3	3	4	4
4	4	5	5	5	5	5	5	5
4	4	2	3	3	3	3	3	3
5	4	4	4	4	4	3	4	3
3	3	3	3	3	2	2	2	3
3	3	3	3	3	2	3	2	3
2	3	1	2	2	2	2	2	2
4	4	4	4	3	3	4	3	4
1	1	4	2	2	2	2	2	3
2	2	2	2	2	2	2	2	2
4	3	3	2	3	2	2	2	3
4	3	3	3	3	2	2	3	3
3	3	2	4	3	3	2	4	3
3	3	4	4	3	3	3	4	3
3	2	2	2	2	2	2	2	2
3	3	5	3	3	3	3	4	3
3	3	4	2	2	2	4	2	3
4	4	3	3	3	3	3	3	3
4	4	2	4	4	2	2	3	3
2	3	2	3	3	2	2	3	3
3	3	3	4	3	2	3	2	3
3	3	4	3	3	2	3	3	3
4	4	3	3	4	4	4	4	3
5	5	3	3	4	3	3	4	3
3	3	3	3	3	3	3	3	3
4	3	2	3	3	3	2	3	4
3	3	3	4	3	3	3	4	4
5	5	3	4	3	2	3	3	4
3	2	3	4	3	2	3	2	4
3	3	3	3	3	3	2	2	3
4	5	2	4	4	4	4	5	5
3	4	4	4	4	2	3	3	4
3	2	3	4	4	4	4	4	4
3	3	3	2	2	2	2	2	3
4	4	4	4	4	4	4	4	4
4	4	4	4	3	2	3	3	4
2	2	3	4	3	3	3	3	3
5	5	5	5	5	5	5	5	5
4	4	4	4	3	4	3	4	4
3	4	2	3	3	2	3	3	3
4	5	5	5	5	5	5	5	5
3	3	3	3	4	3	2	3	3
4	4	2	2	2	2	2	2	3
2	3	2	3	3	2	2	3	2
3	3	3	3	3	3	3	3	3
5	5	4	5	5	5	4	4	3
4	4	3	3	2	3	2	3	2

3	3	4	3	3	3	3	3	3
4	4	4	4	4	4	4	3	3
3	2	2	2	3	2	3	3	2
4	4	3	3	3	3	3	3	2
4	3	2	2	3	2	1	3	3
4	4	5	3	4	3	3	2	3
2	2	3	3	3	3	3	3	3
3	4	2	3	2	1	2	1	2
5	5	3	2	2	2	2	2	4
4	3	2	2	2	2	2	2	2
4	3	3	4	4	5	4	4	4
4	4	4	4	3	2	2	4	3
4	4	4	3	2	3	3	3	4
3	3	3	3	3	2	2	2	3
4	3	3	3	2	2	2	2	2
2	2	3	1	2	2	2	2	2
4	3	3	3	3	3	3	4	3
4	5	4	3	3	4	4	4	4
2	2	3	2	3	2	3	4	3
3	3	3	3	3	3	3	3	3
3	3	5	5	3	2	2	2	3
4	4	3	3	3	3	3	3	3
3	2	3	4	4	4	3	4	4
4	4	3	4	3	2	2	2	2
3	4	4	3	3	4	3	4	3
4	3	4	4	5	4	4	3	4
4	4	2	3	3	3	3	3	3
4	4	4	5	3	3	4	3	3
3	3	3	3	4	2	2	2	4
4	4	4	3	3	4	4	3	4
4	2	4	4	4	4	4	4	4
3	3	3	3	3	3	3	3	3
4	3	2	2	2	2	2	2	2
3	5	3	3	3	2	3	4	3
3	3	2	2	2	2	3	3	4
4	4	5	4	4	4	4	4	4
4	3	2	3	2	2	4	3	3
5	4	3	3	4	3	3	3	3
3	3	2	3	3	3	3	2	3
3	3	4	4	3	3	3	4	2
4	4	2	3	2	2	2	2	2
1	1	1	1	1	1	1	1	2
2	1	2	3	3	3	3	2	3
4	4	3	3	3	3	3	2	3
4	3	2	3	3	3	3	3	3
3	3	3	3	3	3	3	3	3
4	3	3	3	3	2	3	3	3
4	4	3	3	3	4	3	4	4
3	3	5	5	5	1	3	1	5
2	4	4	2	3	3	4	5	3
4	4	4	4	4	4	4	4	4
4	3	3	4	4	4	4	4	4
3	3	3	3	3	3	2	2	3
3	3	2	5	3	2	2	4	3
3	3	2	2	2	2	2	2	2
3	3	4	4	4	5	4	3	2

3	3	3	3	3	3	3	2	3
3	3	1	2	4	2	1	1	2
3	3	3	3	3	3	3	3	3
3	3	3	3	3	3	3	2	3
4	3	3	3	4	3	4	5	3
4	4	4	5	5	5	5	5	2
5	5	5	2	2	2	3	2	2
2	3	2	4	3	1	1	3	3
3	3	3	3	3	3	3	3	2
3	4	2	3	3	2	4	3	3
3	3	3	3	3	3	3	3	3
3	3	3	3	3	3	3	3	3
3	3	3	3	4	4	2	3	4
3	3	3	4	3	3	3	2	2
2	3	3	2	2	1	3	3	3
3	3	4	5	5	3	5	4	4
3	4	2	4	3	4	2	3	2
4	4	3	3	3	3	3	3	3
3	3	2	2	2	2	3	3	3
2	3	2	2	2	2	2	2	2
3	4	3	2	2	4	2	4	2
3	3	3	3	4	4	3	4	3
2	2	3	4	4	4	4	3	2
5	3	3	3	3	3	3	3	4
3	3	3	3	3	2	4	4	4
5	5	5	5	5	3	5	5	4
3	3	2	3	3	2	2	2	2
4	3	3	3	3	2	2	3	2
3	3	3	3	3	3	3	3	3
2	4	3	3	3	2	2	3	3
3	3	3	3	3	3	3	3	3
3	2	2	2	2	2	2	2	2
3	3	3	2	2	2	3	3	2
4	3	4	3	3	3	3	4	4
4	3	3	3	3	2	3	3	3
3	3	3	3	3	3	4	3	3
4	4	2	3	3	3	3	3	3
4	4	4	4	4	4	3	3	4
3	3	3	3	3	3	3	3	3
3	3	3	3	3	3	3	3	3
2	2	2	2	2	2	2	2	2
3	3	5	5	5	5	5	5	4
3	3	2	3	3	2	3	3	3
3	3	3	2	4	4	4	3	3
4	3	3	4	3	3	4	3	4
3	4	2	2	2	2	3	2	2
3	3	3	2	4	2	2	2	3
3	2	2	2	2	2	2	3	3
4	4	4	3	3	3	3	3	4
4	4	3	3	3	3	3	3	3
3	4	3	2	3	3	3	3	3
4	3	2	2	2	2	2	3	2
4	3	3	3	3	3	3	3	3
3	2	2	3	2	2	3	2	3
3	2	1	2	2	2	3	2	2
3	3	2	2	3	2	2	2	2

4	4	2	3	2	2	3	2	3
4	4	3	4	3	2	3	4	2
3	3	3	3	3	3	3	3	3
4	3	2	2	2	2	2	2	3
3	4	4	3	4	3	3	2	3
3	3	5	5	3	4	3	2	2
1	1	4	2	2	1	1	2	3
4	4	2	5	4	3	3	3	2
3	3	3	3	3	3	3	3	3
3	2	2	2	2	2	2	2	3
3	3	2	3	3	3	3	3	3
2	2	3	3	1	1	2	3	2
4	4	4	4	4	4	3	5	4
3	3	2	3	3	3	3	2	3
3	3	4	3	3	3	3	3	3
4	3	3	3	3	1	3	4	3
3	3	3	3	3	3	3	3	3
4	3	4	4	5	4	3	4	3
3	3	4	4	4	3	4	4	3
3	3	2	2	2	3	2	2	2
4	4	5	5	5	5	5	4	4
3	3	4	3	4	3	3	4	4
3	3	3	3	3	3	3	3	3
3	3	4	2	2	2	3	3	3
3	3	3	3	3	4	3	3	3
3	3	2	3	3	2	3	2	3
3	3	3	4	4	2	4	3	4
4	4	4	4	4	4	4	4	3
2	2	2	3	3	2	2	2	2
3	3	4	4	4	4	5	4	4
2	2	3	2	3	3	4	3	2
4	4	3	4	4	3	4	4	4
3	3	4	4	3	3	4	2	2
3	2	4	2	2	2	4	3	2
3	4	3	3	3	3	4	4	4
3	3	3	3	3	3	3	3	3
3	3	3	3	3	4	3	3	3
2	2	3	3	3	2	2	3	3
3	3	5	5	5	4	3	4	4
2	3	3	3	3	3	3	3	3
4	3	4	4	3	4	4	3	4
3	3	3	2	2	2	2	2	2
3	3	3	3	3	3	3	3	3
3	3	3	3	3	3	2	2	3
5	5	5	5	4	4	4	4	5
3	3	3	4	4	3	2	4	3
3	3	4	3	3	2	2	3	3
3	3	3	4	4	4	4	4	3
4	3	3	3	3	3	3	3	3
4	2	2	2	3	3	3	3	3
4	4	3	3	4	3	4	4	4
4	5	3	3	3	4	3	4	3
3	3	3	3	3	3	3	3	3
3	3	3	3	3	3	3	3	3
3	4	3	5	3	2	3	3	3
4	3	4	5	4	3	3	4	3

3	2	1	2	2	2	2	2	3
3	3	3	4	3	3	3	3	3
2	2	2	3	3	1	1	1	2
3	3	2	2	2	2	3	3	2
3	3	3	3	3	3	3	3	3
2	3	3	3	3	3	3	3	3
2	2	3	3	2	2	2	3	2
3	2	3	3	3	3	3	2	3
3	3	4	4	4	4	4	4	3
4	4	4	3	3	3	5	5	4
3	3	3	3	3	3	3	3	2
2	2	3	3	3	3	3	3	3
5	5	5	5	5	5	5	5	5
4	4	4	4	4	4	4	4	4
4	3	4	4	4	4	4	4	3
2	3	3	3	3	3	3	3	3
2	4	3	4	2	5	1	1	2
3	2	3	3	4	2	4	4	4
3	3	3	3	1	5	3	4	3
4	3	4	4	4	2	3	3	2
3	3	2	3	3	3	3	2	3
2	3	4	4	4	4	4	4	4
3	2	3	4	3	3	3	3	3
5	5	4	3	4	4	4	3	4
3	3	5	4	3	3	3	3	3
3	3	3	3	3	3	3	3	3
2	2	3	4	4	3	2	3	3
5	5	4	4	4	4	4	4	4
4	3	4	4	3	4	4	4	4
3	3	3	3	3	3	3	3	3
3	3	4	4	3	4	4	4	4
4	4	4	4	4	4	4	4	4
3	3	3	3	3	3	3	3	3
3	3	3	3	3	2	2	2	2
4	4	4	4	4	4	4	4	4
3	3	2	3	3	3	3	4	4
3	3	3	3	3	3	3	3	3
4	4	4	3	3	3	4	4	3
5	5	5	5	5	5	5	5	5
2	2	5	2	3	4	3	4	4
4	4	5	3	2	3	3	3	3
4	3	4	4	4	4	4	4	4
4	4	4	3	3	3	3	3	3
3	2	3	3	3	3	3	3	3
4	3	3	3	4	3	4	4	5
3	3	3	3	3	3	3	3	3
4	4	3	4	3	4	3	3	3
3	2	3	2	1	1	2	3	3
3	2	3	3	3	3	3	3	2
4	4	4	4	4	4	4	4	4
3	3	3	4	3	1	1	3	3
3	3	3	3	3	4	4	3	4
3	3	3	2	3	2	3	2	3
1	2	5	5	5	5	5	5	5
3	3	3	3	3	3	3	3	3
4	2	2	4	4	4	4	3	3

4	4	3	3	3	3	3	3	3
3	2	2	3	3	2	3	3	3
3	3	3	3	3	2	3	2	2
4	2	3	4	2	2	4	1	4
2	2	2	3	2	2	2	2	3
2	3	2	3	3	3	3	3	3
3	2	1	3	1	1	1	1	2
2	3	3	3	4	3	4	3	3
3	2	3	3	3	3	2	4	3
3	3	2	1	3	1	3	3	3
2	2	2	2	3	2	2	2	3
4	3	3	3	3	3	3	3	3
3	2	2	2	2	2	2	3	2
5	2	2	4	4	4	4	4	2
3	3	3	3	3	3	3	3	3
3	3	3	3	3	2	3	3	3
3	3	3	3	3	2	2	2	3
3	3	3	3	3	3	3	3	3
3	2	2	3	2	2	2	2	1
3	2	3	3	4	2	2	3	4
3	2	5	3	4	3	4	4	3
3	2	3	3	3	3	3	3	3

AIL8	AIL9	AIL10	AIL11	AIL12	AIL13	AIL14	AIL15	AIL16
3	4	2	3	4	2	4	2	2
3	2	2	2	2	2	3	2	3
4	4	3	4	4	4	4	4	4
3	2	2	2	2	2	2	2	1
3	3	3	3	2	3	2	3	4
4	3	4	4	4	4	4	4	4
4	4	4	4	4	4	4	4	4
3	3	3	3	3	3	3	3	3
4	4	4	4	4	4	4	4	4
5	5	5	5	5	5	5	5	5
3	4	3	3	4	2	4	2	4
2	4	4	4	4	4	4	4	4
3	4	3	3	3	3	2	2	3
3	3	3	3	3	3	3	3	3
2	2	2	2	2	2	2	2	1
4	4	3	4	4	4	3	4	4
4	2	3	3	2	2	2	2	2
2	3	2	2	2	2	2	3	3
3	2	3	3	3	3	2	2	2
2	3	3	3	3	2	2	3	3
2	3	3	2	2	4	3	2	1
3	3	3	3	3	3	3	4	3
2	2	2	3	2	2	2	2	2
5	4	4	3	3	3	4	4	4
4	4	2	4	4	2	4	2	2
4	3	3	3	3	3	3	3	4
2	4	3	2	2	4	3	3	2
4	3	2	3	3	2	3	2	2
3	3	2	2	3	4	3	2	2
4	3	3	4	3	3	3	3	3
3	4	3	3	3	3	4	4	4
3	3	3	3	3	3	3	4	3
3	2	2	3	3	3	2	2	5
2	2	3	3	3	3	2	2	2
4	4	3	4	4	3	4	4	3
2	3	3	4	3	4	3	3	3
3	4	2	3	2	2	2	2	4
3	3	2	3	3	3	3	3	3
5	5	5	2	5	4	3	4	4
3	4	3	4	4	4	4	3	3
3	4	4	3	4	4	4	4	4
4	3	2	3	2	2	3	3	3
4	4	4	4	4	4	4	5	4
4	4	4	4	4	4	4	4	3
3	3	3	3	3	3	3	3	3
5	5	5	4	5	4	5	5	5
4	4	4	4	4	4	4	4	4
2	3	2	2	3	3	3	3	2
5	5	5	5	5	5	5	2	5
3	3	3	3	3	3	3	3	3
3	3	3	2	2	3	3	2	1
3	2	2	3	2	4	4	4	2
3	3	3	3	3	3	3	3	3
4	5	4	5	4	4	5	5	5
2	2	3	3	3	3	3	3	3

3	3	3	3	3	3	3	3	3
4	4	3	4	4	4	4	2	2
2	4	3	3	2	4	2	2	3
3	3	3	3	3	3	3	3	3
2	3	2	3	2	3	2	1	1
4	3	2	4	4	4	3	3	3
3	3	3	3	3	3	3	3	3
2	4	2	2	2	4	2	2	2
2	2	4	2	2	4	4	4	4
2	4	2	2	2	2	2	2	2
3	4	4	3	3	4	4	5	4
3	2	3	3	2	3	2	1	3
4	4	3	4	4	2	3	3	3
3	3	2	3	3	3	2	2	2
3	3	5	3	3	4	2	2	2
3	2	2	3	2	2	2	1	1
3	4	3	3	3	3	3	3	3
4	4	4	4	4	4	3	4	4
3	4	3	4	4	4	4	3	2
3	3	3	3	3	3	3	3	3
5	2	3	5	5	5	2	2	2
3	3	3	3	3	3	3	3	3
3	2	4	3	3	2	4	3	2
3	2	3	4	4	4	2	2	2
3	3	2	3	3	2	3	3	4
4	5	4	5	5	5	5	5	4
3	3	3	2	2	3	3	3	3
3	3	4	3	3	4	2	4	2
4	3	4	4	3	4	4	4	4
4	3	4	3	3	4	3	3	5
4	4	4	4	4	4	4	4	4
3	3	3	3	3	3	3	3	3
2	4	3	3	3	2	4	3	3
4	4	3	4	5	3	4	4	3
2	3	2	2	2	2	3	2	4
4	4	4	4	4	4	4	3	4
4	4	4	2	2	4	4	4	4
3	4	3	3	3	2	3	4	4
3	3	2	3	3	3	3	2	2
4	3	4	3	3	3	3	4	3
3	2	3	2	2	3	3	3	2
1	1	1	1	1	1	1	1	2
3	4	3	3	4	3	3	3	3
3	3	3	3	3	3	3	3	3
2	3	3	2	2	3	3	3	3
4	3	3	3	3	3	3	3	3
3	4	3	3	3	3	5	3	3
4	5	3	3	3	4	4	3	4
4	5	5	5	2	5	5	1	1
2	4	2	4	3	4	4	2	4
4	5	4	5	4	4	4	4	4
3	4	4	4	4	4	4	4	4
3	2	2	3	3	3	3	3	3
3	4	4	2	2	5	4	4	4
3	2	2	2	2	2	2	2	2
4	4	4	4	4	4	4	4	5

3	3	3	3	3	3	3	3	3
2	4	2	2	1	1	2	2	2
3	3	3	3	3	3	3	3	3
3	3	3	3	3	3	3	3	2
2	4	2	4	4	4	3	3	3
5	5	5	4	4	5	5	5	5
5	5	3	5	3	2	5	2	2
3	5	3	1	1	5	2	2	2
3	3	3	3	3	3	3	3	3
2	3	3	2	2	2	3	3	3
4	3	3	3	3	3	3	3	3
3	3	3	3	3	3	3	3	3
3	4	3	4	4	3	4	4	4
4	3	3	3	3	4	4	2	2
2	5	2	2	3	4	2	2	2
4	5	5	4	3	4	4	3	4
4	3	4	3	2	2	4	2	2
3	3	3	3	3	3	3	3	3
3	3	3	2	2	3	3	3	3
3	2	4	2	2	2	2	2	2
3	4	2	2	2	2	3	4	4
4	3	4	3	4	4	3	3	4
3	4	2	3	3	4	4	3	3
4	4	3	4	3	3	3	3	4
4	4	4	3	3	4	4	4	2
5	5	2	5	5	5	5	5	5
3	2	2	3	2	3	2	2	2
3	3	2	3	3	4	2	2	2
3	3	2	3	3	3	3	3	3
3	3	3	3	3	3	3	3	3
4	3	3	3	3	3	3	3	3
3	3	2	2	2	3	2	2	2
3	4	3	3	2	3	3	3	3
4	3	4	4	4	4	3	3	3
3	3	2	2	2	3	3	3	3
3	3	3	3	3	3	3	3	3
4	3	3	2	2	2	3	3	3
4	4	4	4	4	4	4	4	4
4	3	3	3	3	3	3	3	3
3	3	3	3	3	3	3	3	3
2	2	3	2	2	3	2	2	2
3	5	3	5	5	5	5	3	3
3	5	5	5	3	3	5	3	3
3	4	2	2	3	3	2	3	3
4	4	3	3	3	4	4	3	4
3	4	2	2	2	2	2	2	2
4	3	2	3	3	2	3	2	2
2	2	2	2	2	2	2	2	2
3	3	3	3	3	3	3	3	3
3	3	3	3	3	3	3	3	3
3	3	3	3	2	2	5	3	3
3	3	2	3	2	2	3	3	3
3	3	3	3	3	3	3	3	3
3	4	2	3	3	4	2	2	3
2	2	2	2	2	2	2	2	3
2	3	2	2	2	2	2	2	2

2	4	2	2	2	4	2	2	2
4	3	3	3	3	3	4	3	3
3	3	3	3	3	3	3	3	3
3	2	2	2	2	2	3	2	2
4	3	4	3	3	4	3	4	3
5	3	3	3	3	5	3	5	3
5	2	3	3	2	2	2	1	1
3	4	3	2	4	4	4	4	4
3	3	4	3	3	3	3	3	3
3	2	2	2	2	2	2	2	2
3	3	3	3	3	3	3	3	3
4	2	4	2	2	3	2	2	2
4	4	3	4	4	4	4	4	4
3	3	3	3	4	4	3	3	3
3	4	3	3	3	3	3	3	3
5	5	3	3	3	4	5	3	3
3	3	3	3	3	3	3	3	3
4	4	4	4	4	3	4	4	4
4	4	3	4	4	3	4	4	4
2	3	3	2	2	2	3	2	3
4	4	3	4	5	5	4	4	4
3	4	4	4	3	4	3	4	4
3	3	3	3	3	3	3	3	3
4	3	3	3	3	2	3	3	3
3	3	3	3	3	3	4	3	3
3	3	4	3	2	3	3	2	2
3	2	4	3	3	3	3	4	4
4	4	3	4	4	4	4	4	3
2	3	3	2	2	3	3	3	2
4	4	4	4	4	4	4	4	4
3	4	2	2	3	2	3	2	2
4	4	4	4	4	4	4	3	4
4	3	4	4	4	4	3	3	3
4	3	4	2	2	2	4	2	2
3	4	4	3	3	4	4	4	4
3	3	3	3	3	3	3	3	3
3	3	4	3	3	2	3	3	3
3	2	3	3	3	3	3	3	2
5	5	5	5	5	4	4	5	4
3	3	3	3	3	3	3	3	3
4	4	3	4	4	4	4	4	3
3	3	3	3	2	2	3	3	3
4	3	3	3	3	3	3	3	3
5	2	2	3	3	3	2	2	2
5	4	4	5	4	4	4	4	4
3	2	3	3	3	4	3	4	4
4	3	3	4	3	3	4	4	4
4	4	4	4	4	4	4	4	4
3	4	3	3	3	3	3	3	3
4	3	3	2	3	3	3	3	3
4	4	3	3	3	3	3	4	3
3	4	3	3	3	3	3	3	3
3	3	3	3	3	3	3	3	3
3	3	3	3	3	3	3	3	3
4	3	3	1	4	4	4	4	4
4	4	5	3	3	5	4	4	4

2	2	2	1	1	2	2	2	2
3	3	3	3	3	3	3	3	3
2	3	1	2	2	3	3	2	2
4	3	2	2	2	2	3	3	3
3	3	3	3	3	3	3	3	3
3	3	3	3	3	3	3	3	3
2	3	2	3	2	3	2	2	2
3	3	3	3	3	4	3	2	3
4	4	4	4	4	4	4	4	4
4	5	4	4	4	5	3	3	3
3	3	3	3	3	3	3	3	3
3	3	3	3	3	3	3	3	3
5	5	5	5	5	5	5	5	5
4	4	4	4	4	4	4	4	4
4	3	4	4	4	4	3	4	4
3	3	3	3	3	3	3	3	3
2	4	5	1	4	1	5	1	3
4	4	5	2	1	3	4	4	4
3	2	2	3	3	1	4	3	3
4	3	4	4	4	4	3	2	2
3	3	3	2	2	3	3	3	3
4	4	4	4	4	4	4	4	4
3	3	3	3	3	3	3	3	3
4	4	4	4	5	5	5	5	5
4	4	4	4	5	3	4	3	4
3	3	3	3	3	3	3	3	3
2	3	3	3	3	3	3	3	3
4	5	4	4	4	4	4	4	4
4	3	4	4	4	4	4	4	4
3	3	3	3	3	3	3	3	3
4	4	4	4	4	4	4	4	4
4	4	4	4	4	4	4	4	4
3	3	3	3	3	3	3	3	3
3	2	2	3	3	3	2	2	2
4	4	4	4	4	4	4	4	4
2	4	4	4	4	3	3	3	4
3	3	3	3	3	3	3	3	3
3	3	4	3	4	4	3	3	4
5	5	5	5	5	5	5	5	5
5	1	3	3	4	3	3	4	3
5	3	3	3	4	4	3	3	3
4	4	4	4	4	4	4	4	4
4	3	4	3	3	3	3	4	3
3	3	3	3	3	3	3	3	3
3	5	3	4	4	4	3	4	4
3	3	3	3	3	3	3	3	3
3	3	3	4	3	4	3	3	3
3	1	2	2	2	2	1	2	3
3	3	3	3	3	3	3	3	3
4	4	4	4	4	4	4	4	4
3	2	3	3	3	3	3	3	3
3	4	2	3	3	3	3	4	3
3	2	3	2	2	3	3	3	3
5	5	5	5	5	5	5	5	5
3	3	3	3	3	3	3	3	3
4	4	3	2	2	2	3	3	3

4	4	3	2	3	4	5	5	3
3	2	3	2	2	4	3	2	3
4	3	2	3	3	3	3	2	2
3	4	2	2	2	4	2	2	4
2	3	2	2	2	3	3	2	2
4	3	4	2	2	3	3	2	3
2	3	1	1	1	3	1	2	2
3	3	3	3	4	3	4	3	4
4	3	3	3	2	3	3	3	3
2	3	3	1	1	3	3	3	3
2	3	2	2	2	3	2	2	2
3	3	3	3	3	3	3	3	3
2	2	2	2	2	2	2	2	2
4	4	4	2	2	2	4	4	5
3	3	3	3	3	3	3	4	4
3	3	3	2	3	4	3	2	4
3	4	2	3	3	3	3	3	2
3	3	3	3	3	3	3	3	3
2	2	3	3	2	3	3	3	1
4	4	4	4	4	3	4	3	3
5	4	3	4	4	4	4	4	4
3	3	3	3	3	3	3	3	3

AIL17	T1-Index	Group	T1-Gender	T1-Grade	T1-Subject	CT1	CT2	CT3
4	1	1	2	4	2	3	3	3
3	2	1	2	4	1	4	3	3
4	3	1	2	4	1	3	3	4
3	4	1	2	4	2	2	1	3
3	5	1	2	3	1	3	3	4
4	6	1	2	3	2	4	2	4
4	7	1	2	3	1	3	5	4
3	8	1	2	2	1	3	2	4
4	9	1	2	3	2	1	3	1
5	10	1	2	3	1	3	4	4
4	11	1	2	3	1	3	3	3
4	12	1	2	4	1	3	3	3
3	13	1	2	2	1	3	3	3
3	14	1	1	3	2	3	2	3
3	15	1	2	4	1	3	4	4
3	16	1	2	4	2	2	3	3
2	17	1	2	3	2	3	3	3
3	18	1	2	3	1	3	3	3
2	19	1	2	3	1	3	3	4
3	20	1	2	3	2	3	5	4
4	21	1	1	3	1	3	4	3
3	22	1	2	3	2	2	3	4
2	23	1	2	4	1	3	2	3
3	24	1	1	2	2	2	2	2
3	25	1	2	4	1	2	2	4
4	26	1	2	4	1	2	2	2
3	27	1	2	3	2	2	2	2
4	28	1	1	4	2	3	3	3
2	29	1	2	2	2	2	2	2
3	30	1	1	1	1	2	2	2
4	31	1	2	1	2	3	3	3
3	32	1	1	1	2	3	1	3
3	33	1	2	1	1	3	3	3
2	34	1	1	3	2	3	3	3
3	35	1	2	1	2	2	3	4
3	36	1	1	1	1	3	4	4
4	37	1	2	3	1	2	3	2
3	38	1	1	3	2	4	3	5
3	39	1	1	3	2	3	3	3
4	40	1	2	4	1	3	4	4
4	41	1	2	2	2	3	4	3
3	42	1	1	4	1	3	3	3
4	43	1	1	2	2	3	3	3
4	44	1	2	2	1	3	3	5
3	45	1	2	3	1	3	3	3
5	46	1	1	3	2	1	2	2
4	47	1	1	4	2	3	3	3
3	48	1	2	4	1	3	3	4
4	49	1	2	3	1	2	3	4
4	50	1	2	1	2	3	4	4
1	51	1	1	1	2	3	3	3
4	52	1	1	3	2	1	2	2
3	53	1	2	3	1	1	4	2
5	54	1	1	1	2	3	4	5
3	55	1	2	3	2	3	3	3

3	56	1	2	1	2	2	3	2
4	57	1	2	1	2	3	1	4
4	58	1	2	3	2	2	3	3
3	59	1	1	3	2	4	4	4
2	60	1	2	1	2	3	1	3
3	61	1	2	1	1	2	2	4
3	62	1	2	1	1	3	4	2
3	63	1	2	3	2	3	3	4
4	64	1	1	1	2	2	1	1
2	65	1	1	3	2	2	2	3
4	66	1	2	3	2	3	3	3
4	67	1	2	1	2	2	2	2
3	68	1	1	1	2	3	3	3
2	69	1	2	3	2	3	2	4
2	70	1	2	1	2	2	3	2
1	71	1	2	2	1	3	4	4
3	72	1	1	2	2	1	1	1
4	73	1	2	2	1	2	4	3
3	74	1	2	2	1	3	3	5
4	75	1	1	2	1	2	3	3
2	76	1	1	2	2	3	2	4
3	77	1	2	3	2	2	3	3
3	78	1	1	3	2	2	2	3
2	79	1	1	3	2	2	2	2
4	80	1	1	3	2	3	3	3
3	81	1	1	3	2	3	3	5
3	82	1	1	3	1	3	2	3
4	83	1	1	3	2	1	1	3
3	84	1	1	3	2	3	3	4
3	85	1	1	3	1	3	3	4
4	86	1	2	3	1	3	3	3
3	87	1	1	3	2	1	1	2
3	88	1	1	3	2	3	3	3
4	89	1	1	3	1	4	4	4
4	90	1	2	1	2	2	1	1
4	91	1	1	3	2	3	3	5
4	92	1	1	2	2	2	3	4
3	93	1	1	3	2	3	3	5
2	94	1	1	3	1	3	3	3
4	95	1	2	2	2	2	2	2
2	96	1	2	3	2	3	4	4
1	97	1	2	3	1	5	2	2
3	98	1	2	2	1	2	2	3
3	99	1	1	3	2	2	2	2
3	100	1	1	2	2	2	3	4
3	101	1	2	1	2	2	4	2
3	102	1	2	3	2	4	4	4
3	103	1	2	3	2	3	4	2
3	104	1	1	3	2	3	3	3
3	105	1	2	1	2	3	3	5
4	106	1	1	3	2	3	3	3
4	107	1	2	1	1	3	2	3
3	108	1	1	3	2	2	5	5
5	109	1	2	3	2	3	3	3
4	110	1	1	3	2	3	1	3
4	111	1	1	1	2	3	3	3

3	112	1	1	3	2	3	4	4
1	113	1	2	1	1	3	3	4
3	114	1	1	1	2	2	3	4
2	115	1	2	1	2	3	3	3
5	116	1	1	3	2	2	3	2
5	117	1	1	3	1	4	4	4
5	118	1	1	1	2	1	1	3
3	119	1	2	3	2	2	3	2
3	120	1	2	1	2	2	1	2
4	121	1	2	4	2	2	3	4
3	122	1	1	4	2	3	5	4
3	123	1	1	3	2	2	3	3
4	124	1	2	1	2	3	3	3
3	125	1	2	1	2	2	3	4
2	126	1	2	3	2	3	3	3
4	127	1	2	3	2	3	3	3
3	128	1	1	1	2	3	3	3
3	129	1	2	3	1	3	3	4
3	130	1	1	1	1	2	1	4
4	131	1	1	3	2	3	3	3
4	132	1	1	1	2	2	3	3
4	133	1	1	3	2	1	1	3
3	134	1	1	1	2	3	3	3
3	135	1	1	3	2	2	3	3
4	136	1	1	3	2	4	3	3
5	137	1	1	3	1	1	3	3
2	138	1	2	1	1	1	2	3
4	139	1	2	1	1	4	3	4
4	140	1	2	3	1	3	2	2
3	141	1	2	3	1	2	2	2
3	142	1	1	1	2	3	3	3
2	143	1	1	3	1	2	3	4
3	144	1	2	1	1	2	3	3
3	145	1	2	1	1	3	3	3
2	146	1	2	3	2	3	3	3
3	147	1	2	1	2	4	4	4
3	148	1	1	3	1	1	3	3
4	149	1	1	3	1	2	4	3
3	150	1	1	1	2	2	3	3
3	151	1	1	1	1	2	2	3
2	152	1	2	3	1	1	2	5
5	153	1	2	3	2	3	5	4
3	154	1	1	1	2	1	3	3
4	155	1	2	3	2	3	3	2
3	156	1	1	3	2	2	2	4
1	157	1	1	1	2	4	4	4
3	158	1	2	3	2	2	2	4
2	159	1	2	3	2	2	3	3
3	160	1	1	3	2	3	4	3
3	161	1	2	1	2	4	3	5
3	162	1	2	1	2	2	2	4
4	163	1	1	3	2	4	5	4
3	164	1	2	3	2	2	3	2
4	165	1	2	1	2	2	3	4
3	166	1	2	3	2	2	3	3
2	167	1	2	3	2	3	3	4

3	168	1	2	1	2	2	3	3
4	169	1	2	1	2	3	3	3
3	170	1	2	1	2	3	3	3
4	171	1	1	1	2	3	4	5
4	172	1	1	2	2	3	3	3
5	173	1	2	3	1	2	1	2
2	174	1	1	3	2	4	4	4
5	175	1	2	2	2	2	2	3
3	176	1	2	2	2	3	3	3
2	177	1	2	2	2	2	3	3
3	178	1	2	1	2	2	3	4
3	179	1	1	2	2	3	3	4
5	180	1	2	1	2	3	3	4
2	181	1	2	2	2	2	3	3
3	182	1	1	2	1	3	2	3
4	183	1	2	2	2	1	4	4
3	184	1	2	2	2	3	3	3
4	185	1	2	2	1	2	2	3
4	186	1	2	2	1	3	2	3
3	187	1	1	2	2	3	4	4
4	188	1	2	2	2	3	3	4
4	189	1	2	2	1	3	4	4
3	190	1	2	2	2	5	5	4
4	191	1	2	2	2	2	3	4
3	192	1	2	2	2	2	2	3
2	193	1	2	2	1	3	4	3
4	194	1	2	2	2	2	3	4
4	195	1	1	2	2	2	3	3
2	196	1	2	2	1	2	3	3
4	197	1	2	2	2	3	5	4
2	198	1	2	2	1	3	3	2
4	199	1	1	2	2	3	3	4
3	200	1	2	2	2	2	3	2
2	201	1	2	2	2	3	2	2
4	202	1	2	2	2	3	3	4
3	203	1	1	3	2	4	4	4
3	204	1	2	2	2	2	3	5
2	205	1	2	2	1	4	4	4
4	206	1	2	1	2	2	3	3
2	207	1	2	2	1	3	4	4
4	208	1	2	2	2	2	2	3
2	209	1	2	2	2	3	4	3
3	210	1	2	2	2	4	3	4
2	211	1	1	2	1	3	3	4
4	212	1	1	2	2	2	2	2
2	213	1	2	2	2	3	3	3
3	214	1	2	2	2	3	4	4
4	215	1	2	2	1	3	2	3
4	216	1	2	2	1	2	4	3
3	217	2	2	3	2	2	3	2
3	218	2	1	2	2	3	3	3
5	219	2	1	2	1	3	3	3
3	220	2	2	3	2	2	3	2
3	221	2	1	2	2	4	4	3
3	222	2	2	2	2	3	4	3
5	223	2	2	2	2	4	4	4

2	224	2	2	3	2	2	2	4
4	225	2	2	2	2	3	3	3
2	226	2	1	3	1	3	4	3
2	227	2	2	4	2	5	5	5
3	228	2	1	2	1	5	4	4
3	229	2	2	3	2	3	4	2
3	230	2	2	3	2	5	5	5
3	231	2	1	2	1	3	3	3
4	232	2	2	4	1	3	3	3
2	233	2	1	2	1	3	4	3
3	234	2	1	2	1	3	3	3
3	235	2	1	2	1	5	5	5
5	236	2	2	2	1	3	3	2
4	237	2	1	2	1	4	4	4
4	238	2	1	2	2	2	3	2
3	239	2	1	2	2	3	3	3
2	240	2	1	2	1	2	3	2
4	241	2	1	3	2	5	5	4
3	242	2	1	2	2	4	3	4
2	243	2	2	2	1	1	3	3
2	244	2	2	2	1	4	4	4
4	245	2	1	4	1	4	3	4
3	246	2	1	2	2	3	3	3
4	247	2	1	2	1	3	3	3
3	248	2	1	2	1	4	4	4
3	249	2	2	1	1	3	3	3
3	250	2	2	2	2	4	4	4
4								
4								
3								
4								
4								
3								
2								
4								
4								
3								
4								
5								
4								
3								
4								
3								
3								
4								
3								
3								
4								
3								
4								
3								
3								
2								
5								
3								
3								

3
2
3
2
3
3
2
3
4
3
2
3
2
3
3
3
4
3
2
4
5
3

CT4	CT5	CT6	AIL1	AIL2	AIL3	AIL4	AIL5	AIL6
3	3	3	3	3	3	3	3	3
3	3	3	3	3	3	2	2	2
3	3	3	3	3	3	3	3	3
2	3	3	2	2	2	2	2	3
3	4	3	3	4	3	3	3	3
2	2	2	2	2	2	2	2	2
4	4	3	4	4	4	3	3	4
3	2	2	4	4	3	2	3	4
3	4	4	1	1	2	1	3	2
3	3	3	3	3	3	3	3	3
3	3	3	3	3	3	3	3	3
3	2	3	3	3	3	3	3	3
3	3	3	2	3	5	3	3	4
3	3	2	4	3	3	2	2	2
4	4	4	4	4	3	4	4	3
3	3	3	3	3	3	3	3	3
3	3	3	4	4	3	3	3	3
3	4	3	3	3	3	3	3	3
3	4	3	3	2	2	1	1	1
4	4	3	3	3	3	3	3	3
3	3	3	3	3	3	2	2	2
4	4	3	3	3	3	3	3	2
2	2	2	1	3	3	2	2	2
2	3	3	3	3	3	3	3	3
3	3	3	3	4	3	3	4	4
2	2	2	2	2	2	2	2	2
3	3	3	3	3	3	3	3	3
3	2	2	2	2	3	2	2	3
1	2	2	2	2	1	1	1	1
2	2	2	2	4	3	2	2	3
3	3	3	3	3	3	3	3	5
2	4	2	2	4	3	2	3	3
3	3	3	3	3	3	3	3	3
3	3	2	2	3	2	3	3	3
3	3	3	3	5	3	3	2	2
4	4	4	4	4	4	2	3	2
2	3	3	1	3	2	2	2	2
4	5	4	4	4	4	4	4	4
3	3	3	3	3	3	3	3	3
3	3	3	4	4	4	3	4	4
3	3	3	2	2	3	3	3	3
3	3	3	3	3	3	3	3	3
3	3	3	4	2	4	3	3	3
3	4	3	2	2	2	1	1	1
3	4	3	3	3	3	2	2	2
1	3	2	2	3	2	3	3	3
3	3	4	4	4	4	3	3	3
3	3	3	3	3	3	3	3	2
2	2	2	1	1	1	1	2	1
4	4	4	4	4	4	4	4	4
3	3	3	3	3	3	3	3	3
2	3	2	3	3	3	3	3	3
2	4	2	3	3	3	3	3	3
3	4	3	3	3	3	2	3	3
3	4	4	3	3	3	2	2	3

3	3	3	2	2	2	2	2	2
1	2	2	3	5	5	2	4	4
2	3	3	2	3	3	3	3	3
4	3	3	4	4	3	3	3	3
3	4	3	3	2	2	2	2	2
2	3	3	3	3	3	3	3	3
2	2	2	3	3	3	3	3	3
1	3	2	3	3	3	3	3	3
1	1	2	1	2	3	1	3	1
2	4	3	1	3	4	1	1	2
4	4	4	3	4	3	3	2	2
2	3	3	2	3	4	2	2	3
3	3	3	3	3	3	3	3	2
2	4	4	2	2	3	3	3	3
2	2	2	1	2	2	1	1	2
4	4	3	3	3	3	2	2	2
1	2	2	2	2	2	2	2	2
3	2	1	2	2	2	2	2	2
3	3	3	4	4	4	2	3	3
3	3	3	2	4	4	3	3	3
3	2	3	3	3	2	2	2	2
3	3	2	2	3	3	2	2	2
3	1	3	2	3	4	1	1	1
2	2	2	2	4	4	2	3	3
3	3	3	2	3	3	2	3	2
5	3	3	3	3	3	3	3	3
3	3	3	3	3	3	3	3	3
1	3	3	3	3	3	3	3	3
4	4	4	4	4	4	3	3	4
2	3	2	2	3	3	3	2	2
3	3	4	3	4	3	2	3	3
2	2	2	3	3	1	1	1	1
3	3	4	3	3	3	2	2	3
4	4	4	3	4	4	3	3	3
1	2	1	2	2	1	1	1	3
3	3	4	3	4	4	4	3	3
2	2	2	2	3	3	2	2	2
3	3	3	3	4	2	2	2	3
3	3	2	3	3	3	3	3	3
2	2	3	3	2	3	2	3	2
4	4	3	2	2	3	2	3	3
2	2	2	3	3	3	3	3	3
3	3	2	1	1	1	2	2	2
2	2	2	2	3	2	2	2	2
2	2	3	2	3	2	3	3	2
2	4	3	2	3	2	3	2	2
3	3	3	3	3	3	2	2	2
3	5	2	2	3	1	1	3	2
3	3	3	3	3	3	3	3	3
3	3	3	3	4	3	2	3	2
3	3	3	3	3	3	3	3	3
3	3	2	2	3	3	3	3	3
4	4	4	3	5	3	2	3	4
2	4	3	3	3	3	3	3	3
3	3	3	2	4	3	2	2	3
3	3	3	3	3	3	3	3	3

4	4	4	4	4	4	4	2	3
3	3	3	3	3	4	3	3	3
3	4	3	2	5	3	2	3	2
3	3	3	3	3	3	3	4	4
3	3	3	2	3	3	2	2	2
4	4	4	4	4	4	3	2	3
1	2	2	1	1	1	1	1	1
2	3	3	2	2	2	2	4	3
1	4	3	3	3	3	3	3	2
4	3	3	3	4	3	3	3	3
4	4	4	2	2	2	2	2	2
3	3	2	3	2	1	1	2	2
3	3	3	3	3	3	3	3	3
3	3	3	2	3	3	2	3	2
3	3	3	3	3	3	3	3	3
3	3	3	4	3	3	3	3	3
3	3	3	3	3	3	3	3	3
3	3	3	2	2	2	3	2	2
2	2	2	2	4	4	2	2	4
3	3	3	3	3	3	3	3	4
3	3	3	3	3	2	2	3	3
1	3	3	2	3	2	2	2	2
3	3	3	4	3	3	3	4	4
3	2	3	3	3	3	2	3	2
3	3	3	3	2	2	2	3	2
3	4	2	3	4	2	2	2	2
2	4	2	2	1	2	1	2	2
3	4	3	3	2	2	2	3	3
2	4	2	2	2	2	2	2	3
3	3	4	2	3	2	1	2	2
3	2	2	1	1	1	1	1	2
3	4	3	3	3	3	2	2	3
3	4	3	2	5	3	3	2	2
3	3	3	2	3	2	2	2	3
3	3	3	3	2	2	2	2	2
4	4	4	4	4	4	4	4	4
2	3	3	1	1	2	2	2	4
3	2	2	2	3	3	3	2	2
3	3	3	3	2	3	2	2	3
2	3	2	3	3	2	2	3	3
3	3	4	3	1	1	3	4	3
3	4	3	4	4	2	3	4	4
2	2	2	2	2	2	1	1	2
2	3	3	2	3	3	2	3	3
4	2	2	1	3	1	2	2	2
4	4	4	3	4	3	3	3	3
3	3	3	3	3	3	2	3	3
3	3	3	2	3	2	2	2	2
3	3	3	3	3	4	3	3	4
4	4	4	3	3	3	3	3	3
2	3	3	2	4	4	2	4	2
4	4	4	4	4	4	4	4	4
1	3	2	2	4	2	2	2	2
4	3	2	2	4	3	2	2	4
2	2	3	2	2	3	3	2	2
3	4	3	4	3	4	3	2	4

2	3	3	1	1	1	1	1	1
3	2	3	2	3	3	3	3	3
2	3	3	3	3	3	3	3	3
3	4	4	2	3	3	3	3	3
4	1	2	2	3	3	1	1	1
1	1	1	2	2	2	2	2	2
4	4	4	4	4	4	4	4	4
2	3	3	1	2	3	2	2	1
3	3	3	3	3	3	3	3	3
3	3	2	2	3	2	4	2	2
4	3	4	3	2	2	1	3	4
3	2	3	3	2	2	1	1	1
4	4	4	3	3	3	4	3	4
2	3	3	3	3	2	2	2	2
2	3	3	3	3	3	2	3	3
3	3	2	2	3	2	2	1	1
3	3	3	2	2	2	1	2	2
2	2	2	2	2	2	2	3	2
3	3	3	3	3	3	3	3	3
2	3	3	3	4	3	3	2	3
2	3	2	2	3	2	1	2	3
3	2	2	2	2	3	3	3	3
5	5	5	5	5	5	5	5	5
3	4	3	3	3	3	2	2	2
2	2	2	3	2	3	2	2	2
2	3	3	3	2	3	1	3	2
3	3	3	2	3	3	2	2	2
3	3	2	2	3	2	1	2	3
2	3	2	3	4	4	2	2	4
3	3	4	3	3	2	2	2	2
2	3	3	2	3	3	3	3	3
3	3	3	2	2	2	2	2	2
2	3	2	1	1	1	3	2	2
2	3	3	3	3	3	3	3	3
3	3	3	2	3	3	2	2	3
3	3	3	3	2	3	2	3	3
3	1	2	2	2	2	2	2	2
4	4	4	4	3	4	2	3	3
3	3	2	3	3	4	4	4	4
3	3	3	3	2	2	2	2	3
2	3	3	2	2	2	3	2	3
2	3	3	4	3	4	2	3	3
3	3	3	4	4	4	4	4	4
3	4	3	3	3	3	3	3	2
3	2	2	2	4	3	2	3	3
3	3	3	3	3	3	3	3	3
3	3	3	3	3	3	3	3	3
2	2	2	2	3	3	3	2	3
2	2	2	4	3	4	4	3	4
3	3	1	3	3	2	4	3	3
3	3	3	3	3	3	3	3	3
1	1	4	3	3	3	3	3	3
3	2	3	1	1	2	2	2	2
3	3	3	3	3	3	3	3	3
4	3	3	3	3	2	2	3	3
4	4	4	4	4	4	4	4	4

2	3	2	2	3	4	2	3	2
3	3	3	3	3	3	3	3	3
4	5	4	4	3	3	3	3	2
5	5	5	5	5	5	5	5	5
2	5	3	3	3	3	3	3	3
3	4	4	3	4	3	3	3	2
5	5	5	5	5	5	5	5	5
3	3	3	3	3	3	3	3	3
3	3	3	3	3	3	2	4	4
3	3	3	3	3	3	3	3	3
3	3	3	3	3	3	3	3	3
5	5	5	5	5	5	5	5	5
2	2	3	3	2	2	2	3	3
4	4	4	4	4	4	4	4	4
2	2	3	2	2	2	2	2	2
3	3	3	4	4	4	4	4	4
3	3	3	4	1	2	3	4	4
4	3	3	2	2	2	2	2	5
3	3	3	2	4	4	3	4	4
3	3	3	3	3	3	3	3	3
4	4	4	4	4	4	4	4	4
5	5	5	5	5	5	5	5	5
3	3	3	3	3	3	3	4	5
3	3	3	3	3	3	3	3	3
4	4	3	4	4	4	4	3	4
3	3	3	3	3	3	3	3	3
4	4	4	4	4	4	4	4	4

AIL7	AIL8	AIL9	AIL10	AIL11	AIL12	AIL13	AIL14	AIL15
3	3	3	2	2	2	2	2	2
2	3	3	3	3	2	3	3	3
4	3	4	3	4	4	3	3	2
3	3	3	2	3	3	3	3	3
3	3	3	4	4	3	3	3	4
4	2	4	2	2	2	2	3	3
4	4	4	4	4	4	4	4	4
3	3	4	3	2	4	2	3	4
2	1	2	2	2	2	2	1	2
3	3	3	3	3	3	3	3	3
3	4	4	3	4	4	4	4	4
3	3	3	3	3	3	3	3	3
3	5	5	5	5	4	5	3	2
2	3	3	3	3	3	2	2	2
3	3	4	3	3	3	3	3	3
3	3	3	3	3	3	3	3	3
3	3	3	3	3	3	3	3	3
3	3	3	3	3	3	3	3	3
1	1	2	1	1	2	1	2	3
3	3	4	3	3	3	3	3	4
2	2	2	2	2	2	2	2	2
2	2	2	2	3	3	3	3	3
2	1	2	2	2	2	4	2	2
3	3	3	3	3	3	3	3	4
2	2	2	2	2	2	4	2	2
2	2	2	2	2	2	2	2	2
3	3	3	3	3	3	3	3	3
4	3	4	2	3	4	3	3	2
1	2	1	1	1	1	4	1	1
3	2	3	2	2	2	3	2	2
3	3	3	3	3	3	5	4	2
4	3	4	3	3	4	4	3	2
3	3	3	3	3	3	3	3	3
2	3	2	3	3	3	3	3	3
3	2	2	2	2	2	2	2	2
3	4	4	4	3	4	4	4	4
2	2	3	2	2	2	3	2	2
4	4	4	4	4	4	4	4	4
3	3	3	3	3	3	3	3	3
4	4	4	4	4	4	4	4	4
3	3	3	3	2	2	2	3	2
3	3	3	3	3	3	3	3	3
3	3	4	3	4	4	4	4	4
1	1	1	1	1	1	1	1	1
3	2	3	3	3	2	3	3	3
2	3	3	3	3	3	3	3	2
4	3	3	3	3	3	3	4	4
3	2	3	3	3	3	3	3	3
2	1	3	2	2	2	1	2	2
4	4	4	4	4	4	3	4	4
3	3	3	3	3	3	3	3	3
3	3	3	3	3	3	3	3	3
3	3	3	2	2	2	3	3	3
3	3	3	3	3	3	3	3	2
2	3	2	3	3	3	3	3	3

2	3	4	4	3	2	2	2	2
2	4	4	3	4	3	4	4	3
3	3	3	3	3	3	3	3	3
3	3	3	3	3	3	3	2	3
2	2	2	2	2	2	2	2	2
3	3	3	3	3	3	3	3	3
2	2	2	2	2	2	2	3	3
3	3	3	3	3	3	3	3	3
1	1	4	4	2	2	3	1	1
2	2	3	2	2	2	3	2	2
4	3	3	3	4	3	3	2	3
3	3	4	3	3	4	4	4	3
3	3	3	3	3	3	3	3	3
3	3	3	3	3	3	3	3	3
2	2	2	2	1	1	2	2	1
2	2	2	2	3	2	2	2	2
2	2	2	2	2	2	2	2	2
2	2	2	2	2	2	2	2	2
2	2	3	3	4	4	4	4	3
3	3	3	3	3	3	3	3	3
4	2	3	2	2	2	2	2	2
3	2	3	3	2	2	3	2	2
1	1	4	2	1	1	3	1	1
2	2	2	2	2	2	3	2	2
2	2	2	2	2	2	3	2	2
3	3	3	3	3	3	3	3	3
3	3	3	3	3	3	3	3	3
3	3	3	3	3	3	3	3	3
2	3	4	2	3	3	4	3	3
2	2	2	3	2	2	2	4	4
3	3	3	3	3	2	3	3	3
2	1	1	1	1	1	1	1	1
3	3	3	3	3	3	3	3	3
3	3	3	3	3	3	3	3	3
3	3	1	1	1	1	1	1	1
3	2	4	2	2	2	3	2	2
2	3	3	3	3	3	3	3	3
2	2	4	3	2	2	2	2	2
3	4	3	3	3	3	3	3	3
3	3	4	3	3	3	3	3	1
3	3	3	3	3	3	3	3	3
3	3	3	3	3	3	3	3	4
2	3	4	2	1	2	2	1	2
3	2	3	3	2	2	3	2	3
2	2	3	2	2	2	2	3	2
4	2	5	4	4	4	5	2	2
3	3	3	3	3	3	4	3	4
2	2	2	4	2	2	3	2	2
3	3	3	3	3	3	3	3	3
4	4	3	3	3	3	3	2	3
3	3	3	3	3	3	3	3	3
3	3	3	3	3	3	3	3	2
4	2	4	1	3	3	3	1	1
3	4	3	3	4	3	3	3	3
3	4	2	2	2	3	3	3	2
3	3	3	3	3	3	3	3	3

4	3	3	4	4	4	3	3	3
3	4	4	3	3	3	4	3	3
2	2	2	2	2	2	2	2	2
3	3	4	3	3	3	4	3	3
2	2	3	2	3	3	3	2	3
2	2	4	3	2	3	4	3	4
2	1	1	1	1	1	2	2	2
3	3	3	3	3	3	2	2	2
3	3	4	3	3	3	4	3	4
3	3	3	3	4	3	3	3	3
1	4	4	4	3	3	3	3	3
2	2	2	2	3	2	3	2	2
3	3	3	3	3	3	3	3	3
2	3	3	3	3	3	3	3	2
3	3	3	3	3	3	3	3	3
3	3	3	3	3	3	3	3	3
3	3	3	3	3	3	3	3	3
3	4	4	2	3	2	2	3	4
2	4	4	4	2	2	4	4	3
3	2	3	3	3	3	3	3	3
4	3	4	2	4	3	3	3	2
2	2	3	3	2	1	3	2	2
4	3	4	4	3	3	4	3	3
4	3	3	3	3	2	4	4	4
3	3	3	3	3	2	3	2	2
3	3	3	2	2	2	2	2	3
1	4	3	2	3	2	1	1	1
3	4	3	3	4	2	3	3	2
2	2	2	2	2	2	2	2	2
2	3	2	2	2	2	4	2	2
1	2	3	2	2	3	2	2	2
2	2	2	2	3	2	3	3	3
2	2	3	2	2	2	3	3	2
2	2	3	2	2	2	3	2	2
3	2	4	2	2	3	3	3	3
4	4	4	4	4	4	4	4	4
2	2	3	2	3	2	3	2	3
2	2	3	3	2	2	4	2	2
3	4	3	3	4	3	3	3	4
3	3	3	3	2	2	3	2	2
4	5	3	3	3	3	5	4	3
1	5	4	2	3	3	4	3	2
1	2	2	2	2	2	2	2	2
3	2	3	2	3	2	2	3	3
2	2	2	2	4	2	2	2	2
3	3	4	3	3	3	3	3	3
3	2	4	2	2	2	2	2	2
4	3	3	3	3	3	4	3	3
4	3	3	3	3	3	3	2	3
3	3	4	3	3	3	3	3	3
2	2	3	2	3	3	3	3	3
4	4	4	4	4	4	4	4	4
2	2	3	2	2	2	3	2	2
3	3	3	3	3	2	4	2	2
2	3	2	2	2	2	4	2	1
3	3	3	3	3	3	4	3	3

1	1	3	2	2	3	4	2	3
3	4	3	3	3	4	4	3	3
3	3	3	3	3	3	3	3	3
3	3	4	2	3	2	3	3	3
3	3	3	3	3	2	2	3	3
2	2	2	2	2	2	2	2	2
3	4	4	4	4	4	4	4	4
1	2	2	2	2	1	2	2	2
2	2	3	2	2	2	3	2	2
4	3	4	3	3	3	2	2	3
2	3	4	3	3	3	2	3	3
1	1	2	1	1	1	1	1	2
4	3	4	3	4	4	4	4	3
2	2	2	2	2	2	3	2	3
2	3	2	3	3	2	3	2	3
1	1	1	2	1	1	2	1	1
3	3	4	2	2	1	4	3	3
2	2	3	2	2	2	3	3	3
3	3	3	3	3	3	4	2	3
3	3	4	2	3	2	3	3	2
2	2	2	2	2	1	3	2	1
4	4	3	3	3	3	3	3	3
5	5	5	5	5	5	5	5	5
2	2	2	3	3	2	4	2	2
3	3	3	2	2	2	2	3	2
2	3	3	1	2	2	2	3	1
3	3	3	3	2	3	2	3	2
2	3	3	2	3	3	4	3	2
3	2	4	2	3	2	4	3	2
2	3	3	3	2	2	2	2	4
2	3	3	3	3	3	3	3	2
2	2	2	2	2	2	2	2	2
3	2	1	1	1	1	1	1	1
3	3	3	3	3	3	3	3	3
3	3	4	4	3	2	4	2	4
3	2	3	2	3	2	3	2	3
3	2	2	2	2	2	2	2	2
5	4	4	4	5	3	3	3	2
4	4	2	2	3	3	3	3	3
2	2	3	2	3	2	4	3	3
3	3	3	3	3	2	3	2	2
3	3	3	3	4	2	4	4	3
4	4	4	4	4	4	4	4	4
3	3	4	3	3	3	3	3	3
3	3	4	3	3	3	4	3	3
3	3	3	3	3	3	3	3	3
3	3	3	3	3	3	3	3	3
3	2	2	3	2	2	4	3	2
3	3	4	4	3	4	3	3	3
3	4	3	3	3	4	2	3	4
3	3	3	3	3	3	3	3	3
3	3	3	3	2	5	4	3	3
2	2	3	3	3	3	3	3	4
3	3	3	3	3	3	3	3	3
2	2	2	2	3	2	2	2	2
4	4	4	4	4	4	4	4	4

2	2	3	4	3	3	3	4	4
3	3	3	3	3	3	3	3	3
3	3	3	2	3	3	4	3	3
5	5	5	5	5	5	5	5	5
3	3	3	3	2	2	3	4	3
2	3	3	3	4	3	4	4	3
5	5	5	5	5	5	5	5	5
3	3	3	3	3	3	3	3	3
4	4	5	4	4	4	4	3	4
3	3	4	3	3	3	3	3	3
3	3	3	3	3	3	3	3	3
5	5	5	5	5	5	5	5	5
3	3	3	3	3	3	3	3	2
4	4	4	4	4	4	4	4	4
2	2	2	2	2	2	2	2	2
4	4	4	3	3	3	4	4	3
1	3	3	3	3	3	4	4	3
3	3	3	3	3	3	3	4	4
3	4	2	2	3	3	3	3	2
3	3	3	3	3	3	3	3	3
4	4	4	4	4	4	4	4	4
5	5	5	5	5	5	5	5	5
4	5	5	4	3	3	5	5	5
3	3	3	3	3	3	3	3	3
4	3	4	4	4	3	3	4	3
3	3	3	3	3	3	3	3	3
4	4	4	4	4	4	4	4	4

AIL16	AIL17
2	2
2	3
2	4
3	3
3	4
4	2
4	5
3	2
2	2
3	3
3	4
3	3
2	3
2	4
3	3
3	3
3	3
3	3
3	2
3	3
2	3
3	3
2	2
4	3
4	2
2	2
3	3
3	3
1	1
2	2
4	4
4	4
3	3
3	3
2	3
4	4
2	2
4	4
3	3
4	4
2	2
3	4
4	4
1	1
3	3
2	3
4	4
3	3
2	3
4	4
3	3
3	2
4	3
3	4
3	3

3	3
3	4
2	3
3	3
2	2
3	3
2	2
3	3
2	1
3	3
2	4
4	4
3	3
3	3
1	1
2	2
2	3
2	2
3	4
3	3
2	2
2	3
1	1
2	2
2	3
3	3
4	4
3	3
3	4
4	4
3	4
1	3
3	4
3	3
3	3
2	3
3	3
2	4
3	3
1	4
3	3
2	3
2	4
3	2
2	3
3	5
3	4
2	3
3	3
3	3
3	3
2	2
1	1
4	4
2	4
3	3

3	4
3	4
3	3
3	3
2	4
2	3
2	2
2	4
4	3
3	3
3	3
2	3
3	3
3	4
3	3
3	3
3	3
3	4
2	4
2	3
3	4
2	3
4	4
4	4
3	3
1	3
1	4
3	3
2	2
2	3
2	3
3	2
2	4
2	2
3	3
4	4
3	4
2	3
2	3
1	3
4	4
4	5
2	3
2	4
2	4
3	3
2	4
3	3
3	3
2	3
2	2
4	4
2	3
3	3
2	4
3	4

3	1
3	2
3	4
3	3
2	2
2	3
3	4
1	2
2	2
4	3
2	4
1	2
4	4
2	3
3	3
1	3
2	3
4	4
4	3
3	5
2	3
3	3
5	5
2	2
2	2
1	3
2	2
2	4
2	4
4	4
3	2
2	2
1	1
3	3
2	2
2	2
2	2
3	5
3	3
2	2
2	3
3	4
4	4
4	3
3	3
3	3
3	3
2	3
4	3
3	2
3	3
3	3
3	2
3	3
2	2
4	4

2	3
3	3
3	3
5	5
3	3
4	4
5	5
3	3
4	4
3	3
3	3
5	5
3	3
4	4
2	2
3	4
3	3
4	3
4	2
3	3
4	4
5	5
5	5
3	3
4	3
3	3
4	4
	4
