# Supplementary material for: The impact of university students’ computational thinking on AI literacy: A longitudinal study based on SEM-PLS
Source: PLoS One. 2026 May 27;21(5):e0350124. doi: 10.1371/journal.pone.0350124 (PMC13215501; doi:10.1371/journal.pone.0350124)
Supplement: S1 Table — (DOCX) [file pone.0350124.s002.docx]

S2 Table. English Questionnaire

| Constructs | Items | Sources |
| --- | --- | --- |
| CT | I admire people who can demonstrate a clear stance when making decisions. | (Korkmaz et al. ,2017) |
|  | I admire people who can remain objective when thinking about problems. |  |
|  | I believe that with enough time and effort, I can solve most of the problems I face. |  |
|  | When encountering new situations, I believe I can solve the problems that may arise. |  |
|  | I believe I can develop targeted solutions for the problems I encounter and solve them. |  |
|  | Dreams can motivate me to achieve my most important goals. |  |
|  | When solving problems, I trust my intuition about what is "right" and "wrong." |  |
|  | When handling multiple tasks, if I encounter a problem with the current task, I will switch to other tasks and come back to solve it later. |  |
|  | When facing a problem, I can quickly construct a mathematical model to solve it. |  |
|  | I believe I am particularly interested in mathematical processes. |  |
|  | With the help of mathematical symbols and concepts, I think I can better learn programming instructions. |  |
|  | I believe I can easily capture the relationships between numbers. |  |
|  | I can solve problems encountered in daily life using mathematical methods. |  |
|  | I can translate verbally expressed mathematical problems into digital form. |  |
|  | I enjoy experiencing collaborative learning with other members of my group. |  |
|  | In collaborative learning, I believe the team's collective effort leads to more satisfactory results. |  |
|  | I enjoy working with members in collaborative learning to solve problems related to group projects. |  |
|  | I think collaborative learning generates more ideas. |  |
|  | When facing complex problems, I can formulate and execute effective solution strategies. |  |
|  | I find it enjoyable to try and solve complex problems. |  |
|  | I am willing to learn challenging things. |  |
|  | I take pride in being able to think very precisely. |  |
|  | When comparing various options and making decisions, I use a systematic method. |  |
|  | I find it difficult to convey my problem solutions to others. |  |
|  | My problem is knowing where and how to use variables (like X and Y) to solve problems. |  |
|  | I am unable to apply the solutions I have developed. |  |
|  | When faced with a problem, I find it difficult to come up with multiple solutions. |  |
|  | In a collaborative learning environment, I am unable to form my own ideas. |  |
|  | In collaborative learning, learning new knowledge with my group members makes me feel tired. |  |
| AIL | I can identify whether the software I use is supported by artificial intelligence. | (Laupichler et al. ,2023) |
|  | I can give examples of software supported by artificial intelligence. |  |
|  | I can explain the difference between humans and artificial intelligence. |  |
|  | I can explain the difference between General AI and Narrow AI. |  |
|  | I can evaluate whether media portrayals of AI (e.g., in movies or video games) exceed the current capabilities of AI technology. |  |
|  | I can list the disadvantages of artificial intelligence. |  |
|  | I can list the advantages of artificial intelligence. |  |
|  | I can describe the potential risks associated with using artificial intelligence. |  |
|  | I can describe the potential benefits associated with using artificial intelligence. |  |
|  | I can describe the potential future impact of artificial intelligence. |  |
|  | I can distinguish between existing AI technologies and undeveloped AI technologies. |  |
|  | I can explain how AI applications make decisions. |  |
|  | I can explain in general terms how machine learning works. |  |
|  | I can describe how a machine learning model is trained, validated, and tested. |  |
|  | I can explain the difference between supervised and unsupervised learning. |  |
|  | I can explain how reinforcement learning works. |  |
|  | I can explain the relationship between deep learning and machine learning. |  |
|  | I can explain the meaning of the term "Artificial Neural Network." |  |
|  | I can critically evaluate the outcomes of AI applications in at least one disciplinary field. |  |
|  | I can explain why data plays an important role in the development and application of AI. |  |
|  | I can describe why humans play an important role in the development of AI systems. |  |
|  | I can describe how an AI system acts and reacts within a defined environment. |  |
|  | I can explain how computers use sensors to collect data that can be used for AI purposes. |  |
|  | I can list applications that use AI for natural language processing/understanding. |  |
|  | I can identify ethical issues surrounding artificial intelligence. |  |
|  | I can explain the meaning of the term "black box" in AI systems. |  |
|  | I can describe how bias can arise in AI systems. |  |
|  | I can critically reflect on the potential impact of AI on individuals and society. |  |
|  | I can explain why artificial intelligence is becoming increasingly important. |  |
|  | I can explain the difference between rule-based systems and machine learning systems. |  |
|  | I can assess which problems within my field of expertise are suitable for AI methods and which are not. |  |
|  | I can describe what artificial intelligence is. |  |
|  | I can describe the concept of Explainable AI (XAI). |  |
|  | I can clearly articulate the necessity of data security when using AI applications and explain the importance of data privacy. |  |
|  | I can describe the concept of big data. |  |
|  | I can give examples of where I might encounter AI in my daily life (personal or professional). |  |
|  | I can explain what an algorithm is. |  |
|  | I am aware of potential legal issues that may arise when using artificial intelligence. |  |

S2 Table. Chinese Questionnaire

| 量表 | 题目 |
| --- | --- |
| 计算思维 | 我欣赏那些在做出决策时能够展现出明确立场的人。 |
|  | 我欣赏那些在思考问题时能够保持客观的人。 |
|  | 我相信如果我有足够的时间和努力，可以解决我所面临的大部分问题。 |
|  | 当遇到新的情况时，我相信我能解决可能出现的问题。 |
|  | 我相信，我能够为遇到的问题制定针对性解决方案，并解决问题。 |
|  | 梦想能促使我实现最重要的目标。 |
|  | 当解决问题时，我相信自己对“正确”和“错误”的直觉。 |
|  | 在处理多个任务时，如果当前任务遇到问题，我会先处理其他任务，之后再回来解决它。 |
|  | 在面对问题时，我能迅速构建数学模型来解决。 |
|  | 我认为，我对数学过程特别感兴趣。 |
|  | 在数学符号和概念的帮助下，我认为我可以更好地学习编程指令。 |
|  | 我相信，我可以轻松地捕捉数字之间的关系。 |
|  | 我可以用数学方式解决在日常生活中遇到的问题。 |
|  | 我可以将口头表达的数学问题数字化。 |
|  | 我喜欢和我的小组其他成员一起体验合作学习。 |
|  | 在合作学习中，我认为团队的共同努力让我将会获得更为满意的成果。 |
|  | 我喜欢在合作学习中与成员一起解决与小组项目有关的问题。 |
|  | 我认为合作学习会产生更多的想法。 |
|  | 在面对复杂问题时，我能制定并执行有效的解决策略。 |
|  | 我认为尝试解决复杂的问题是很有趣的事情。 |
|  | 我愿意学习有挑战性的事情。 |
|  | 我为能够非常精确地思考而感到自豪。 |
|  | 在比较各种选择并做出决定时，我会使用一种系统的方法。 |
|  | 我发现，向他人传递我的问题解决方案很困难。 |
|  | 我的问题是我应该在哪里以及如何使用变量(如X和Y)来解决问题。 |
|  | 我无法应用我所制定的解决方案。 |
|  | 面对问题时，我发现自己难以想出多种解决方案。 |
|  | 在合作学习的环境中，我无法形成自己的想法。 |
|  | 在合作学习中，与我的小组成员一起学习新知识让我感到很累。 |
| 人工智能素养 | 我能分辨出我使用的软件是否得到人工智能的支持。 |
|  | 我能举出得到人工智能支持的软件的例子。 |
|  | 我能解释人类和人工智能之间的区别。 |
|  | 我能解释广义人工智能和狭义人工智能之间的区别。 |
|  | 我能评估人工智能的媒体表现（例如，在电影或视频游戏中）是否超出了人工智能技术的当前能力。 |
|  | 我能说出人工智能的缺点。 |
|  | 我能说出人工智能的优点。 |
|  | 我能描述使用人工智能可能出现的风险。 |
|  | 我能描述使用人工智能可能带来的好处。 |
|  | 我能描述人工智能对未来的潜在影响。 |
|  | 我能够区分现有的人工智能技术与未开发的人工智能技术。 |
|  | 我能解释人工智能应用程序是如何做出决策的。 |
|  | 我能从总体上解释机器学习是如何工作的。 |
|  | 我能描述机器学习模型是如何被训练、验证和测试的。 |
|  | 我能够解释监督学习与非监督学习的区别。 |
|  | 我能够解释强化学习是如何工作的。 |
|  | 我能解释深度学习与机器学习的关系。 |
|  | 我能解释“人工神经网络”这一术语的含义。 |
|  | 我能够对至少一个学科领域的人工智能应用成果做出批判性评估。 |
|  | 我能解释为什么数据在人工智能的开发和应用中发挥着重要作用。 |
|  | 我能描述为什么人类在人工智能系统的发展中发挥着重要作用。 |
|  | 我能描述人工智能系统如何在设定的环境中行动和对环境做出反应。 |
|  | 我能解释计算机如何使用传感器来收集可用于人工智能目的的数据。 |
|  | 我能列举使用人工智能辅助自然语言处理/理解的应用程序。 |
|  | 我能够识别围绕人工智能的伦理问题。 |
|  | 我能解释"黑盒 "一词在人工智能系统中的含义。 |
|  | 我能描述人工智能系统中偏见是如何产生。 |
|  | 我可以批判性地反思人工智能对个人和社会的潜在影响。 |
|  | 我能解释为什么人工智能变得越来越重要。 |
|  | 我能解释基于规则的系统与机器学习系统的区别。 |
|  | 我能够评估在我的专业领域内，哪些问题适合使用人工智能方法来解决，哪些问题不适合。 |
|  | 我能描述什么是人工智能。 |
|  | 我能描述可解释人工智能（Explainable AI）的概念。 |
|  | 我能够清楚地在使用人工智能应用程序时数据安全的必要性，并解释数据隐私的重要性。 |
|  | 我能描述大数据的概念。 |
|  | 我能举出我日常生活（个人或职业）中可能接触人工智能的例子。 |
|  | 我能解释什么是算法。 |
|  | 我知道在使用人工智能时可能出现的潜在法律问题。 |
