# Supplementary material for: The impact of university students’ computational thinking on AI literacy: A longitudinal study based on SEM-PLS
Source: PLoS One. 2026 May 27;21(5):e0350124. doi: 10.1371/journal.pone.0350124 (PMC13215501; doi:10.1371/journal.pone.0350124)
Supplement: Attachment — Submitted filename: Response_to_Reviewers.docx [file pone.0350124.s004.docx]

Subject: Rebuttal Letter: Manuscript PONE-D-25-21913

Dear Dr. Wang and Reviewers,

Thank you for giving us the opportunity to revise our manuscript entitled “The Impact of University Students’ Computational Thinking on AI Literacy: A Longitudinal Study Based on SEM-PLS” (Manuscript ID: PONE-D-25-21913). We greatly appreciate the time and effort that you and the reviewers have dedicated to providing insightful and constructive comments on our manuscript.

We have carefully considered all the comments and have made extensive revisions to the manuscript accordingly. We believe that these revisions have significantly improved the quality and clarity of our manuscript.

Below, we provide a point-by-point response to all the comments raised by the reviewers. All changes in the manuscript have been highlighted in blue for your convenience.

Responses to Reviewers and Editors

(Original comments by reviewers are in blue, modified responses are marked in red, and the specific revised content is presented in black font.)

# **Editor**

## **Comment 1:**

Please ensure that your manuscript meets PLOS ONE's style requirements, including those for file naming. The PLOS ONE style templates can be found at

https://journals.plos.org/plosone/s/file?id=wjVg/PLOSOne_formatting_sample_main_body.pdf and

[https://journals.plos.org/plosone/s/file?id=ba62/PLOSOne_formatting_sample_title_authors_affiliations.pdf](https://track.editorialmanager.com/CL0/https:%2F%2Fjournals.plos.org%2Fplosone%2Fs%2Ffile%3Fid=ba62%2FPLOSOne_formatting_sample_title_authors_affiliations.pdf/1/010f0198eb573164-49e288a7-d978-472a-a8de-b479a725ab36-000000/ZeJlD4Ph8X6Q2Jo1IN_-RKENYAORjQLQiFZdGybPwMY=226)

## **Response 1:**

We have thoroughly reviewed and revised the manuscript format in accordance with the journal's provided style template, and it now fully complies with PLOS ONE's formatting requirements.

## **Comment 2:**

We are unable to open your Supporting Information file [Supporting information.rar]. Please kindly revise as necessary and re-upload.

## **Response 2:**

We sincerely apologize for the inconvenience caused by the format of the previously uploaded compressed file. The Supporting Information files have now been re-uploaded in a .zip archive to ensure they can be properly accessed.

## **Comment 3:**

Please include captions for your Supporting Information files at the end of your manuscript, and update any in-text citations to match accordingly. Please see our Supporting Information guidelines for more information: <http://journals.plos.org/plosone/s/supporting-information>.

## **Response 3:**

We have added detailed descriptions of all supporting information files in the 'Supporting Information' section at the end of the manuscript. Additionally, we have verified that all in-text citations correctly correspond to these files.

## **Comment 4:**

If the reviewer comments include a recommendation to cite specific previously published works, please review and evaluate these publications to determine whether they are relevant and should be cited. There is no requirement to cite these works unless the editor has indicated otherwise.

## **Response 4:**

We have carefully reviewed the recommended references provided by the reviewers. The literature [https://doi.org/10.1007/s40299-021-00570-0] is highly relevant to the data cleaning methods adopted in this study and has been cited in the “Research Methods” section (page 29) to provide a stronger theoretical foundation for this procedure. The reference [https://doi.org/10.1186/s41239-023-00403-8] offers valuable insights into the role of potential external moderating factors and has been incorporated into the “Discussion” section (page 23) to support the analysis of variables that may influence the results. Furthermore, the literature [https://doi.org/10.1080/0144929X.2022.2072768] provides critical support for clarifying the limitations of this study and has been correspondingly introduced in the “Limitations” subsection under the “Discussion” chapter (page 27).

We sincerely appreciate the highly valuable suggestions from the editor and reviewers, which have significantly enhanced the rigor and completeness of this research.

# **Reviewer #1**

## **Comment 1:**

Conceptual introduction: The core variables should be introduced in the first chapter, including at least their brief/plain-language definitions, why they are important, and how they can be connected with each other.

## **Response 1:**

Thank you for your valuable feedback. We fully agree that a more robust theoretical foundation would significantly enhance the quality and depth of this study. In response to your comments, we have revised the Introduction section as follows:

First, following the background information presented in the opening paragraph, we have explicitly incorporated the definition of "AI literacy" in the third sentence, breaking it down into four distinct dimensions. This modification can be found in lines 49–50 on page 2 through lines 53–54 on page 3 of the manuscript.

Second, within the third paragraph, we have expanded the discussion on computational thinking. The opening sentence of this paragraph now emphasizes the importance of computational thinking, followed by a clarification of its definition and dimensional structure. We further elaborate that stronger computational thinking abilities enable individuals to better understand and apply AI-related concepts, thereby fostering the development of their AI literacy. These revisions are located between lines 94 and 102 on page 5.

The revised Introduction now demonstrates a clearer structure, improved logical flow, and strengthened theoretical support, which collectively enhance the overall coherence and persuasiveness of our argument.

The overall revised Introduction is provided below:

The breakthrough development of generative artificial intelligence (GenAI) technologies—exemplified by advanced systems such as DeepSeek—has triggered a cognitive revolution in education, fundamentally reshaping the underlying logic of knowledge transmission [1]. This deep integration of technological innovation and educational paradigms has given rise to a pivotal concept: Artificial Intelligence Literacy (AIL). Long, Magerko (2) defined it as "a set of competencies that enable individuals to critically evaluate AI technologies, communicate and collaborate effectively with AI, and utilize AI as a tool across online, domestic, and workplace contexts." This literacy is widely recognized as comprising four dimensions: awareness, application, evaluation, and ethics [3]. A study published in Nature Methods underscores the growing importance of developing AI literacy [4]. In response, leading academic institutions such as Stanford University and the University of Oxford have begun formulating policies on the use of generative AI tools—including ChatGPT, Bard, DALL·E, and Stable Diffusion—emphasizing the ethical and appropriate use of these technologies within academic settings. In 2019, the United Nations Educational, Scientific, and Cultural Organization (UNESCO) officially established a global educational framework for artificial intelligence literacy in the Beijing Consensus on Artificial Intelligence and Education. In response, China actively initiated corresponding actions. In 2021, the China Educational Technology Center issued the Framework for Students’ AI Technology and Engineering Literacy, which, as an official standard, delineates a matrix of core competencies that students are expected to develop in order to adapt to the demands of the intelligent era. In June 2024, Zhejiang University released the *Red Paper on University Students’ AI Literacy (2024 Edition)*, offering an academic reference for AI literacy in higher education. Subsequently, between October and November, Beijing and Chongqing issued their respective Guidelines for the Application of Artificial Intelligence in Education. These documents not only established a three-dimensional competency framework encompassing “standards–scenarios–skills,” but also emphasized the development of general education courses in generative AI. This initiative aims to systematically enhance students’ adaptability and creativity in an increasingly intelligent and digital society [5]. The formulation of these policies is far from coincidental. Empirical evidence from Ng, Maduranayagam (6) indicates a significant gap in the normative use of AI tools within academia. Enhancing AI literacy goes beyond mere technical understanding of artificial intelligence; it also entails a critical awareness of the ethical and societal implications that arise from its application.

Scholarly research on AI literacy has primarily focused on primary and secondary school teachers, students, and undergraduates. For instance, Ng, Su (7) conducted a comprehensive review of AI literacy education in secondary schools, identifying commonly used instructional methods, tools, content, assessment strategies, and measurement dimensions. Building on a more precise definition of target populations, the academic community has gradually developed a paradigm for measuring AI literacy and attributing its influencing factors. Regarding research methodology, scholars have primarily employed structural equation modeling, individual interviews, and survey questionnaires. For example, Du, Sun (8) adopted a two-step structural equation modeling approach to analyze the data, revealing that K–12 teachers’ perceptions of using AI for social good and their self-efficacy in learning AI are two direct determinants of their behavioral intention to engage in AI learning. Although such quantitative studies have identified the direct determinants of behavioral intention, there remains a lack of systematic exploration into the underlying mechanisms that shape AI literacy.

Computational thinking is an essential key skill for university students [9]. It refers to the application of computer science principles and fundamental concepts for problem-solving and system design, encompassing a range of cognitive activities that involve thinking, understanding, and addressing problems in the manner of a computer scientist [10]. Korkmaz, Çakir (11) proposed that computational thinking skills consist of five dimensions: algorithmic thinking, collaboration, creativity, critical thinking, and problem-solving. Individuals with strong computational thinking abilities are more likely to be proficient in comprehending and utilizing AI-related concepts [12]. Celik (13), in a comprehensive literature review, highlighted gaps in the field of AI literacy research, particularly the insufficient exploration of its influencing factors. Specifically, through empirical analysis, the author proposed a three-dimensional analytical framework encompassing the digital divide, computational thinking, and cognitive absorption, and confirmed the significant positive impact of computational thinking on AI literacy. However, the study remained at the level of validating correlations between variables, without delving into the magnitude or dynamic evolution of computational thinking's effect on AI literacy. From the perspective of research paradigms, current studies on AI literacy are predominantly cross-sectional, with relatively few longitudinal investigations. Research examining the dynamic influence of variables such as computational thinking on AI literacy remains particularly scarce.

This study aims to investigate the impact of computational thinking on the development of AI literacy. A dynamic PLS-SEM model was constructed based on cross-sectional and longitudinal data to systematically analyze the relationship between computational thinking and AI literacy over time to achieve this goal. Accordingly, the study proposes the following research questions:

a) Does computational thinking have a significant impact on AI literacy?
b) Does the influence of computational thinking on AI literacy change over time?
c) Does computational thinking at an earlier time point predict AI literacy at a later stage?

This study presents two key innovations. First, it establishes a close link between computational thinking and AI literacy by examining whether computational thinking, as a critical variable, significantly influences AI literacy. Second, beyond a detailed cross-sectional analysis, the study incorporates a two-month longitudinal data tracking approach to uncover how computational thinking at the initial time point (T0) affects AI literacy at a subsequent time point (T1).

## **Comment 2:**

Rewrite the literature review to emphasize research gaps: The current chapter of the literature review is a series of documented previous studies, with very clear topic sentences. The problem is that the authors overemphasized what is known to us. I believe you should analyze and summarize the research gaps that drive you to your research questions and aims.

## **Response 2:**

Thank you for your valuable comments regarding the literature review section. We have thoroughly revised this part in accordance with your suggestions.

In the rewritten version, we have reorganized the paragraph structure to follow a more logical progression: we first synthesize and critically evaluate existing relevant studies, and then identify key gaps and limitations within the current research field. The corresponding revisions can be found from line 131 on page 6 to line 179 on page 8 of the manuscript.

The revised literature review is presented below:

**Literature Review**

The concept of AI literacy was initially proposed by Kandlhofer, Steinbauer (14), referring to the ability to understand and apply the technologies and concepts underlying artificial intelligence and computer science. This includes knowledge in areas such as graphs and data structures, sorting, search problem-solving, and machine learning. Long, Magerko (2) further defined it as “a set of competencies that enable individuals to critically evaluate AI technologies, communicate and collaborate effectively with AI, and utilize AI as a tool across online, domestic, and workplace contexts.” Wang, Rau (3) expanded this construct by proposing a widely recognized framework consisting of four dimensions: awareness, application, evaluation, and ethics.

Existing research has primarily focused on K–12 teachers and students, as well as undergraduate populations. For instance, Kong, Man-Yin Cheung (15) conducted a study with university students in Hong Kong and found that short-term courses significantly enhanced their AI literacy and empowerment while reducing disciplinary and gender disparities. In a systematic review of AI literacy education in secondary schools, Ng, Su (7) summarized prevalent instructional methods, tools, and assessment strategies. Nevertheless, current studies remain largely confined to specific educational stages, with a noticeable lack of cross-sectional and cross-cultural comparative analyses. Furthermore, there is limited longitudinal research investigating the developmental mechanisms of AI literacy.

Computational thinking (CT) is defined as a set of cognitive activities involving problem-solving and system design through the use of fundamental computer science concepts [10]. A framework proposed by Korkmaz, Çakir (11), which includes five dimensions—algorithmic thinking, collaboration, creativity, critical thinking, and problem-solving—has been widely adopted. Studies indicate that learning programming languages such as Scratch significantly enhances CT skills among primary school students [16]. Additionally, factors such as gender stereotypes, learning motivation, and collaborative learning have been shown to substantially influence CT development [17, 18]. Research participants have ranged from preschoolers to pre-service teachers. For example, a longitudinal study by Zhang and Wong (19) on primary students identified gender, prior computer experience, and formal learning opportunities as significant predictors of CT competency. Similarly, Sun, You (20) found that various background factors influence the computational thinking abilities of STEAM teachers in China. Although diverse methodologies such as structural equation modeling (SEM), meta-analysis, and quasi-experimental designs have been employed [21, 22], most studies remain cross-sectional, making it difficult to infer causal pathways or long-term developmental trajectories of computational thinking.

In terms of research methods, studies on AI literacy often adopt mixed-method approaches incorporating structural equation modeling, interviews, questionnaires, and focus groups. For instance, Zhao, Wu (23) used SEM to elucidate the structural relationships among various dimensions of AI literacy. Du, Sun (8) identified teachers’ self-efficacy and perceived social utility as key factors influencing their willingness to learn AI. Through qualitative analysis, Velander, Taiye (24) highlighted that the often incidental nature of teachers’ AI knowledge acquisition may lead to misunderstandings. Despite these methodological advances, there is a notable scarcity of longitudinal studies that examine AI literacy and computational thinking in an integrated manner. The interaction between these two constructs remains poorly understood and warrants further empirical investigation.

## **Comment 3:**

Could you please add theoretical rationales for CT to predict next-time AIL, and not in the reverse direction? E.g., AIL may be promoted through specific training and technology use, which should subsequently benefit CT skills. Then, why is your hypothesized direction conceptually sound?

## **Response 3:**

Thank you for your insightful comments. You rightly pointed out that AI literacy (AIll) can be cultivated through targeted training and technological application, which in turn may also foster the development of computational thinking (CT) skills. We agree that the relationship between these two constructs is likely bidirectional.

However, this study posits the hypothesis that “computational thinking predicts subsequent AI literacy, but not the reverse,” primarily due to conceptual hierarchical differences between the two. Computational thinking is defined as “the thought processes involved in problem-solving” [25] and represents a set of general cognitive attitudes and skills applicable across various domains [10]. Its core components—such as problem decomposition, pattern recognition, abstraction, and algorithm design—provide a foundational basis for navigating complex and dynamic AI environments [26].

We argue that computational thinking constitutes an essential cognitive framework for meaningful interaction with AI technologies. Proficiency in core CT components (e.g., decomposition, pattern recognition, abstraction, and algorithmic reasoning) enables learners to more effectively understand, utilize, and critically evaluate AI systems. Thus, although AI literacy can indeed be enhanced through specific training, we hypothesize that stronger computational thinking skills facilitate more efficient acquisition and transfer of AI-related knowledge and competencies.

To strengthen the theoretical grounding of this hypothesis, we have supplemented the discussion in the relevant section of the manuscript (lines 181–190, pages 8–9) with additional literature support, framing computational thinking as a precursor to AI literacy.

The overall revised Introduction is provided below:

Computational Thinking (CT) refers to the cognitive processes involved in formulating problems and designing solutions through a series of structured steps [25]. It represents a universally applicable set of attitudes and skills [10]. Competency in problem-solving—a core component of CT—is essential across all disciplines, including computer science and engineering, and is increasingly critical in all domains of human endeavor [26]. Furthermore, CT encompasses problem-solving, critical thinking, and decision-making abilities, which are indispensable for navigating the complex and dynamic landscape of artificial intelligence [26]. It provides a foundational framework for reasoning that enhances both the understanding and development of AI systems. Empirical evidence from Lin, Zhou (27) confirms that computational thinking significantly predicts AI literacy among university students. Celik (13) explored the factors influencing AI literacy among university students and found a positive correlation between students' computational thinking and their level of AI literacy. Greenwald, Leitner (28) emphasized the importance of leveraging concepts such as computational thinking to help students better understand AI knowledge and address AI-related challenges. Similarly, Kim, Jang (29) identified computational thinking as essential for acquiring AI literacy. Based on these findings, the present study hypothesizes that the stronger university students’ computational thinking abilities are, the more conscious, evaluative, and ethically aware they will be when engaging with AI technologies. Accordingly, this study proposes the following hypothesis:

H1: At Time 0 (T0), university students’ computational thinking significantly affects their AI literacy.

H2: At Time 1 (T1), university students’ computational thinking significantly improves their AI literacy.

H3: Computational thinking at Time 0 (T0) significantly and positively predicts AI literacy at Time 1 (T1) among university students.

## **Comment 4:**

Please provide references for your data cleaning process. E.g., <https://doi.org/10.1007/s40299-021-00570-0>

## **Response 4:**

Thank you for providing the key reference regarding the data cleaning procedures. Following your recommendation, we have now supplemented and refined the description of our data cleaning process and criteria with greater detail. Multiple additional references have also been incorporated to methodologically support these revisions. The specific modifications can be found from line 260 on page 12 to line 274 on page 13 of the manuscript.

The specific revised content is provided below:

After completing the necessary preparations, data were collected from 301 undergraduate students at a university. To ensure sample quality, two screening criteria were applied based on established methodologies in the literature [30-33]. The time taken to complete the questionnaire served as one benchmark, while uniform responses (e.g., all identical answers) were treated as indicative of inattention or careless responding [31, 33, 34]. Based on these references, the following criteria were used to filter valid responses: (1) The questionnaire was designed to take approximately 2–5 minutes to complete. Participants who finished in less than 90 seconds were considered to have rushed through the survey irresponsibly, and their data were excluded. (2) Questionnaires with uniform responses (e.g., all “1” or all “5”) or extreme values were also excluded. (3) All IP addresses were recorded and checked for duplicates; repeated entries were removed. After the initial screening, 12 invalid responses were excluded, resulting in 289 valid questionnaires (S1 Table).

## **Comment 5:**

In the research model, a number of potential moderators are not mentioned, e.g., AI use experience, academic achievements, and gender. Is this gender bias common, and why? How much do you estimate it should influence or bias your results? You may check similar research findings and contexts like https://doi.org/10.1186/s41239-023-00403-8 Potential moderators should be discussed in your implications and limitations.

## **Response 5:**

We fully agree with your perspective and have accordingly incorporated a dedicated subsection entitled “Potential Influence of External Factors” into the Discussion section (located between lines 481–499 on pages 23–24) to further elaborate on this matter. The added passage specifically includes the following elements:

1. **Acknowledgment of study limitations:** We explicitly state that the current study has not fully accounted for potential moderating variables such as gender, prior experience with AI, and academic achievement.
2. **In-depth discussion on the potential role of gender:** Drawing upon the recommended reference Lai, Ye (18)as well as other relevant studies [15, 17], we analyze how gender differences may exert moderating effects in the development of both AI literacy and computational thinking. Potential underlying mechanisms—such as learning motivation, engagement, and access to resources—are also discussed.
3. **Suggestions for future research:** We recommend that subsequent studies incorporate these variables more systematically into their analytical frameworks to uncover more comprehensively the interplay between AI literacy and computational thinking.

We believe this addition significantly strengthens the critical examination of the study’s limitations, enhances the depth of the discussion, and improves the overall rigor of the paper. We sincerely thank you for your highly constructive feedback, which has been instrumental in refining this research.

The specific revised content is provided below:

**Discussion of External Factors**

This study, while examining the relationship between AI literacy and computational thinking, did not fully account for the potential influence of external moderating variables such as gender, prior AI experience, and academic achievement. The role of gender warrants particular attention, as existing research indicates that gender differences may play a significant role in the development and manifestation of both AI literacy and computational thinking. For instance, Kong, Man-Yin Cheung (15) found that short-term AI courses significantly reduced disparities in AI literacy and self-efficacy across genders and academic backgrounds, suggesting that gender may serve as an important moderating variable. Similarly, Master, Tang (17) highlighted that student motivation and gender stereotypes significantly influence the development of computational thinking, while Lai, Ye (18) further suggested that collaborative learning can help mitigate gaps related to gender and prior experience, promoting more equitable learning outcomes. These findings imply that gender and other background factors may indirectly moderate the relationship between AI literacy and computational thinking by influencing learning motivation, engagement, and access to resources. Therefore, future research should more systematically incorporate these variables to develop a more comprehensive understanding of the interactive mechanisms between AI literacy and computational thinking.

## **Comment 6:**

Conceptual synthesis: We currently know that AIL and CT are compound concepts, meaning that they have several subdimensions. However, they are treated as high-level variables, ignoring the specific aspects. This is also a limitation of your study. E.g., https://doi.org/10.1080/0144929X.2022.2072768

## **Response 6:**

Thank you for your insightful comments and valuable suggestions. You rightly pointed out that by treating Artificial Intelligence Literacy (AIL) and Computational Thinking (CT) as higher-order holistic constructs, this study did not sufficiently explore the specific relationships among their sub-dimensions—an approach that indeed constitutes a limitation. We fully agree with your assessment and have addressed this point in the “Research Limitations and Future Directions” section of the manuscript (specifically, from line 561 to 568 on page 27, and line 583 to 594 on page 28).

Specifically, we have explicitly stated that, although modeling AIL and CT as aggregate constructs helps capture their broad-level relationship, it does not allow for uncovering the potentially differentiated patterns of association among sub-dimensions such as algorithmic thinking, decomposition, abstraction, and critical thinking within CT, or among understanding of technical concepts, application skills, and ethical reasoning within AIL.

In future studies, we aim to develop a more granular analytical framework that incorporates factors such as the digital divide and perceived trust, employing multi-dimensional instruments for measurement. This will enable a thorough examination of the mechanisms through which these variables interact with each sub-dimension of AIL, as well as how these relationships may vary across different demographic groups. Furthermore, we plan to conduct more focused analyses—such as cross-lagged modeling at the sub-dimensional level and tests of interaction effects with external variables—to deepen the understanding of the internal structure and developmental dynamics between computational thinking and artificial intelligence literacy.

Your feedback has significantly enhanced the rigor of this study and the clarity of its future directions. We once again extend our sincere gratitude for your highly constructive review.

The specific revised content is provided below:

**Research Limitations and Future Directions**

When assessing the level of artificial intelligence literacy, this study considered computational thinking as the sole dependent variable, neglecting other potentially significant influencing factors—such as the digital divide—which somewhat limits the comprehensiveness of the research. Furthermore, both computational thinking and AI literacy were measured as higher-order composite constructs, without examining the nuanced relationships between their specific sub-dimensions (e.g., algorithmic thinking and critical thinking within computational thinking; awareness, evaluation, and ethical reasoning within AI literacy). Although this approach facilitates macro-level analysis, it may obscure distinct relational patterns among subcomponents. Although a longitudinal design was adopted, this study remains correlational in nature and lacks experimental manipulation. As a result, it does not support strong causal inferences. The observed relationships may be influenced by unmeasured variables, such as learning motivation, prior programming experience, or learning environment. Future research could incorporate experimental or quasi-experimental methods, along with more fine-grained dimensional analysis, to further clarify the causal mechanisms and internal structure between AI literacy and computational thinking. Regarding the sample, the gender distribution was imbalanced, with a male-to-female ratio of approximately 2:3, which may restrict the generalizability of the results to broader populations. Although a longitudinal data collection method was employed, only two months may not be sufficient to capture the complex dynamics and long-term developmental trends in the relationships among variables.

To address the limitations identified, this study proposes the following directions for future work: First, to comprehensively investigate AI literacy, future research will incorporate multiple factors—such as the digital divide, perceived trust, and other relevant variables—into an integrated analytical model. A multidimensional questionnaire will be designed to systematically examine the mechanisms through which these factors interact with AI literacy, as well as their differential effects across diverse demographic groups. This approach aims to yield more holistic and nuanced insights. Additionally, greater attention will be devoted to analyzing cross-lagged relationships between sub-dimensions (e.g., algorithmic thinking, critical thinking, ethical evaluation) and their interactions with contextual variables. Such fine-grained analysis will help elucidate the internal structure and dynamic linkages between computational thinking and AI literacy. Second, to enhance the representativeness and generalizability of the results, the sample size will be expanded, with particular emphasis on achieving a balanced gender ratio of 1:1. By ensuring gender diversity within the sample, the study will be better positioned to accurately reflect the realities of AI literacy across different gender groups, thereby improving the applicability and reliability of the findings. Finally, to more effectively capture dynamic changes over time, the study will increase the frequency of data collection and extend the period to three months. This will allow for a more nuanced observation and analysis of how key variables evolve and interact over time, providing a stronger empirical foundation for understanding the developmental patterns of AI literacy.

# **Reviewer #2**

## **Comment 1:**

Temporal Design Validity: The two-month interval between data collection points appears insufficient for robust longitudinal analysis. As the authors acknowledge, "two months may not be sufficient to capture the complex dynamics and long-term developmental trends in the relationships among variables." However, no empirical justification is provided for this timeframe choice. Evidence demonstrating that this duration is adequate to avoid test-retest effects and capture meaningful developmental changes in both computational thinking and AI literacy is needed.

## **Response 1:**

Thank you for raising this important point regarding the time interval selected for data collection in our study. We fully understand your concern about whether a two-month interval is sufficient to support valid longitudinal analysis and whether this decision requires more vigorous empirical justification.

In response to your suggestion, we have expanded the explanation for our choice of time interval in the “Participants” subsection within the “Research Methods” section (lines 236–243 on page 11), providing both theoretical and empirical support. The added content is structured as follows:

First, we clarify the rationale behind the two-month interval between the two measurement points. This decision was informed by methodological recommendations from prior research. For instance, Wang and Zhang (35) suggest that the optimal time lag for detecting mediation effects should be approximately half of the peak time for the indirect effect to emerge, thereby balancing the sensitivity to dynamic changes while minimizing memory effects. This approach is also supported by recent empirical studies in related fields [36].

Second, we elaborate on the relationship between time intervals and observable effects. Drawing on the work of Zhao, Shipp (37), we highlight that the interval length and observed effect size often exhibit an “inverted U-shaped” relationship—intervals that are too short may introduce retest effects or fail to capture meaningful change. At the same time, intervals that are too long may lead to effect attenuation or the omission of critical developmental processes. The two-month interval used in this study was determined based on this theoretical framework and is expected to fall within the ascending segment of the curve, thereby allowing for the sensitive detection of dynamic relationships between computational thinking and AI literacy, while also controlling for common methodological biases.

Lastly, we acknowledge that this time span may still present limitations in revealing long-term developmental trajectories between the variables. Future studies could adopt a multi-wave longitudinal design to more comprehensively and finely delineate the developmental pathways and mechanisms linking these constructs.

Your feedback has significantly enhanced the rigor and persuasiveness of the methodology section. We once again extend our sincere gratitude for your valuable suggestions.

The specific revised content is provided below:

The survey was conducted on the Questionnaire Star platform on September 10 and November 10, 2024, with a two-month interval between the two measurement points. This duration was determined based on methodological recommendations from Wang and Zhang (35), who suggested using half of the peak time for indirect effects, and was further supported by Wu, Li (36). Although a longer interval might better capture long-term changes, Zhao, Shipp (37) demonstrated an inverted U-shaped relationship between time intervals and observable effect sizes. The two-month interval adopted in this study is expected to fall within the ascending phase of this curve, thereby enabling sensitive detection of the dynamic relationship between computational thinking and AI literacy.

## **Comment 2:**

Gender Distribution Imbalance: The sample exhibits a significant gender imbalance with a male-to-female ratio of approximately 2:3 (39% male, 61% female). As noted by the authors, this "may restrict the generalizability of the results to broader populations." Gender-specific analyses should be conducted to ensure no systematic gender differences affect the structural relationships between computational thinking and AI literacy.

## **Response 2:**

Thank you for raising this important point regarding the gender distribution imbalance in our sample. We fully understand your concern that the gender ratio may affect the generalizability of the findings and the structural relationships within the model.

In response to your suggestion, we conducted a multi-group analysis to examine whether the core structural model remains invariant across gender groups. The results of this analysis have been added to the "Participants" subsection under "Research Methods" (see lines 292-297 on page 14 of the manuscript).

As shown in Table 1, the analysis revealed no statistically significant differences in the core path coefficients between gender groups, with all p-values exceeding the .05 significance threshold. These results indicate that the core structural relationships between computational thinking and AI literacy remain consistent across genders, despite the uneven sample distribution. Therefore, the gender imbalance does not appear to introduce systematic bias into the core conclusions of this study, and the findings maintain considerable representativeness and explanatory power.

Table 1 Results of Multi-Group Analysis by Gender

| **Path** | **2-tailed p value** | **Invariance Supported** |
| --- | --- | --- |
| T0 Computational Thinking → T0 AI Literacy | 0.313 | Yes |
| T0 Computational Thinking → T1 AI Literacy | 0.483 | Yes |
| T1 Computational Thinking → T1 AI Literacy | 0.534 | Yes |

The specific revised content is provided below:

Among the 216 valid responses, 84 were from male participants and 132 from female participants. A multi-group analysis was conducted to examine gender invariance across key model pathways (T0-Computation thinking→T0-AI Literacy: two-tailed p = 0.313; T0-Computation thinking→T1-AI Literacy: two-tailed p = 0.483; T1-Computation thinking→T1-AI Literacy: two-tailed p = 0.534). The results indicate that the core model is invariant across genders, supporting the structural stability of the hypothesized relationships and the representativeness of the sample.

## **Comment 3:**

AI Literacy Measurement Transparency: Given that AI literacy concepts are rapidly evolving, full disclosure of the 38-item scale adapted from Laupichler, Aster (29) in the supplementary materials is essential for replication and validity assessment.

## **Response 3:**

Thank you for your valuable suggestion regarding the transparency of the Artificial Intelligence Literacy scale. We fully agree that, given the rapidly evolving nature of the AI literacy concept, full disclosure of the measurement instrument is essential to ensure research reproducibility and facilitate validity assessment.

In response to your recommendation, we have implemented the following improvements:

On one hand, we have submitted the complete 38-item AI literacy scale, adapted from Laupichler, Aster (38), as supplementary material.

On the other hand, in the “Measures” subsection under the “Research Methods” section of the manuscript (line 307, page 14), we have added a clear statement indicating that “S2 Table” thereby enabling readers and future researchers to access and examine the instrument easily.

Your comment has significantly enhanced the transparency and rigor of this study. We sincerely appreciate your guidance.

The specific revised content is provided below:

AI literacy was measured using a scale adapted from the study by Laupichler, Aster (38), which is specifically designed to assess university students’ AI literacy. The scale consists of 38 items (e.g., “I can identify ethical issues surrounding artificial intelligence”) and has been empirically validated in subsequent research by Laupichler, Aster (39) (S2 Table).

## **Comment 4:**

Cross-sectional vs. Longitudinal Findings Discrepancy: The rejection of H3 (β = 0.021, p = 0.708) presents a critical contradiction requiring deeper exploration. While cross-sectional analyses demonstrate that "computational thinking significantly and positively predicts AI literacy" (H1: β = 0.507, H2: β = 0.567, both p < 0.001), the longitudinal predictive relationship from T0 computational thinking to T1 AI literacy is non-significant. This discrepancy undermines the core premise that computational thinking serves as a longitudinal predictor of AI literacy development. The authors must provide theoretical explanations for why cross-sectional associations do not translate to longitudinal predictions, particularly when both constructs show significant improvement over time (H4 and H5 supported).

## **Response 4:**

Thank you for your insightful observations and valuable suggestions regarding the apparent discrepancy between the cross-sectional and longitudinal analytical results. We fully agree that this key contradiction warrants in-depth theoretical interpretation.

In response to your recommendation, we have added a dedicated paragraph to the "Discussion" section (lines 428–461, pages 21–22) to elaborate on this finding. The added content explores the results from the following two theoretical perspectives:

On one hand, the combined influence of the learning context and multiple factors is considered. While cross-sectional relationships reflect static correlations between skills at a single time point, the lack of longitudinal predictive power may stem from the complexity of real educational settings. Over the two-month learning intervention, the development of students' artificial intelligence literacy (AIL) was influenced not only by their initial computational thinking (CT) levels but also by factors such as curriculum progression, project-based practice, peer interaction, and instructor guidance. These rich learning experiences may have diluted or obscured the independent predictive effect of CT in the longitudinal analysis.

On the other hand, potential limitations at the measurement and sample levels are discussed. The relatively high baseline CT scores at T0 may have restricted the predictive variability for subsequent AIL growth. Furthermore, although the AIL measurement tool demonstrated good reliability and validity in cross-sectional contexts, its sensitivity to subtle changes over a short-term intervention might be limited, potentially affecting its ability to accurately capture true developmental changes in longitudinal analyses.

In summary, we argue that this seemingly contradictory result highlights the complexity of the relationship between CT and AIL: the cross-sectional findings emphasize their close cognitive-structural association, while the longitudinal analysis underscores the dynamic, multi-factor moderated nature of AIL development in authentic educational environments.

Your feedback has significantly enhanced the depth and theoretical rigor of this study. We sincerely appreciate your insightful guidance.

The specific revised content is provided below:

An important and intriguing finding of this study is that, while cross-sectional analysis revealed a significant positive correlation between computational thinking (CT) and artificial intelligence literacy (AIL) (supporting H1 and H2), longitudinal analysis showed that T0 CT did not significantly predict T1 AIL (failing to support H3). This result highlights the complexity of the relationship between CT and AIL, warranting in-depth discussion. Firstly, cross-sectional relationships reflect covariation between skills at a single point in time, whereas longitudinal relationships test predictive validity. The cross-sectional findings indicate that students with stronger CT skills at a given moment also tend to possess higher levels of AIL. This covariation may stem from project-based learning or problem-solving tasks, where cognitive processes inherent in CT—such as abstraction, decomposition, and algorithmic reasoning—are simultaneously activated and reinforced alongside the competencies required to understand and apply AI technologies, thereby creating a synergistic effect [40, 41]. However, in the two-month learning intervention of this study, the development of students’ AIL was likely influenced not only by CT but also by multiple other factors. These include the progressive delivery of course content, diverse project-based practices, peer interactions during collaborative activities, and targeted guidance from instructors [42, 43]. These rich learning experiences may have diluted or obscured the independent predictive power of CT over time, resulting in its non-significant longitudinal effect. This interpretation aligns with existing research suggesting that the transfer effect of CT may depend on specific learning contexts and task supports [44]. Secondly, potential ceiling effects or issues with measurement sensitivity may have played a role. The relatively high baseline CT scores at T0 may have limited the scope for further predictive growth in AIL. Moreover, although the AIL assessment tool demonstrated good reliability and validity in cross-sectional contexts, it may lack sufficient sensitivity to detect subtle changes longitudinally—especially in the absence of more differentiated task-based scenarios [45]—and within the short duration of the intervention [46]. In conclusion, the relationship between CT and AIL cannot be fully captured by a simple linear predictive model. The cross-sectional results emphasize the close cognitive connection between the two constructs, while the longitudinal findings suggest that, in authentic educational environments, the development of AIL is moderated by multiple factors including instructional context, student background, and curriculum design [47, 48].

## **Comment 5:**

Causal Inference Limitations: This observational study design precludes strong causal claims. The limitations section should explicitly state that causal relationships cannot be definitively established without experimental manipulation. Additionally, the exclusive focus on computational thinking as a predictor overlooks other potentially important variables mentioned in the literature, including "digital divide, perceived trust," learning environment, individual motivation, and prior programming experience.

## **Response 5:**

Thank you for your important feedback regarding the limitations in causal inference and the potential omission of other predictive variables in this study. We fully agree that, as an observational study, our research design cannot establish strict causal relationships, and the exclusive focus on computational thinking may have overlooked other key influencing factors.

In accordance with your suggestions, we have made significant additions to the Discussion section, which include the following two aspects:

First, we have explicitly clarified the limitations in causal inference (lines 560–576, page 27). We now state: "Although this study adopted a longitudinal design, it remains correlational in nature and did not incorporate active experimental manipulation. Therefore, the observed relationships between variables cannot be interpreted as evidence of definitive causal effects; these associations may still be subject to confounding by unmeasured variables, such as individual learning motivation, prior programming experience, specific learning environments, and perceived trust." This statement directly addresses your concern regarding the need to explicitly acknowledge causal limitations.

Second, we have expanded the discussion on omitted variables and outlined future research directions (lines 480–498, pages 23–24). A new paragraph has been added to emphasize that while this study primarily focused on the predictive role of computational thinking, it may not have sufficiently incorporated other significant factors. Existing research indicates that variables such as the digital divide, perceived trust, quality of learning environments, individual motivation, and prior knowledge and experience also play crucial roles in the development of AI literacy. Future studies should develop theoretical models that include these diverse variables and employ more comprehensive measurement approaches and designs to systematically uncover the multiple mechanisms driving AI literacy development.

Your comments have greatly helped us to more comprehensively and rigorously define and articulate the boundaries and theoretical contributions of this study. We once again extend our sincere gratitude for your insightful review.

The specific revised content is provided below:

**Research Limitations and Future Directions**

When assessing the level of artificial intelligence literacy, this study considered computational thinking as the sole dependent variable, neglecting other potentially significant influencing factors—such as the digital divide—which somewhat limits the comprehensiveness of the research. Furthermore, both computational thinking and AI literacy were measured as higher-order composite constructs, without examining the nuanced relationships between their specific sub-dimensions (e.g., algorithmic thinking and critical thinking within computational thinking; awareness, evaluation, and ethical reasoning within AI literacy). Although this approach facilitates macro-level analysis, it may obscure distinct relational patterns among subcomponents. Although a longitudinal design was adopted, this study remains correlational in nature and lacks experimental manipulation. As a result, it does not support strong causal inferences. The observed relationships may be influenced by unmeasured variables, such as learning motivation, prior programming experience, or learning environment. Future research could incorporate experimental or quasi-experimental methods, along with more fine-grained dimensional analysis, to further clarify the causal mechanisms and internal structure between AI literacy and computational thinking. Regarding the sample, the gender distribution was imbalanced, with a male-to-female ratio of approximately 2:3, which may restrict the generalizability of the results to broader populations. Although a longitudinal data collection method was employed, only two months may not be sufficient to capture the complex dynamics and long-term developmental trends in the relationships among variables.

To address the limitations identified, this study proposes the following directions for future work: First, to comprehensively investigate AI literacy, future research will incorporate multiple factors—such as the digital divide, perceived trust, and other relevant variables—into an integrated analytical model. A multidimensional questionnaire will be designed to systematically examine the mechanisms through which these factors interact with AI literacy, as well as their differential effects across diverse demographic groups. This approach aims to yield more holistic and nuanced insights. Additionally, greater attention will be devoted to analyzing cross-lagged relationships between sub-dimensions (e.g., algorithmic thinking, critical thinking, ethical evaluation) and their interactions with contextual variables. Such fine-grained analysis will help elucidate the internal structure and dynamic linkages between computational thinking and AI literacy. Second, to enhance the representativeness and generalizability of the results, the sample size will be expanded, with particular emphasis on achieving a balanced gender ratio of 1:1. By ensuring gender diversity within the sample, the study will be better positioned to accurately reflect the realities of AI literacy across different gender groups, thereby improving the applicability and reliability of the findings. Finally, to more effectively capture dynamic changes over time, the study will increase the frequency of data collection and extend the period to three months. This will allow for a more nuanced observation and analysis of how key variables evolve and interact over time, providing a stronger empirical foundation for understanding the developmental patterns of AI literacy.

**Discussion of External Factors**

This study, while examining the relationship between AI literacy and computational thinking, did not fully account for the potential influence of external moderating variables such as gender, prior AI experience, and academic achievement. The role of gender warrants particular attention, as existing research indicates that gender differences may play a significant role in the development and manifestation of both AI literacy and computational thinking. For instance, Kong, Man-Yin Cheung (15) found that short-term AI courses significantly reduced disparities in AI literacy and self-efficacy across genders and academic backgrounds, suggesting that gender may serve as an important moderating variable. Similarly, Master, Tang (17) highlighted that student motivation and gender stereotypes significantly influence the development of computational thinking, while Lai, Ye (18) further suggested that collaborative learning can help mitigate gaps related to gender and prior experience, promoting more equitable learning outcomes. These findings imply that gender and other background factors may indirectly moderate the relationship between AI literacy and computational thinking by influencing learning motivation, engagement, and access to resources. Therefore, future research should more systematically incorporate these variables to develop a more comprehensive understanding of the interactive mechanisms between AI literacy and computational thinking.

## **Comment 6:**

Attrition Analysis: The study experienced substantial participant attrition from the initial sample of 301 students to the final 216 participants (28.2% attrition rate). Specifically, 39 participants from the initial sample did not complete the second-round questionnaire, and an additional 35 responses were excluded due to invalid completion patterns. However, no systematic analysis was conducted to examine whether participants who dropped out differed systematically from those who remained in terms of baseline computational thinking abilities, AI literacy levels, or demographic characteristics. This lack of attrition analysis limits the ability to assess potential selection bias and threatens the internal validity of the longitudinal findings. A comparison between completers and non-completers on key study variables should be provided to ensure that the observed relationships are not artifacts of differential dropout patterns.

## **Response 6:**

Thank you for your important feedback regarding participant attrition. We fully understand your concern that the attrition rate may introduce selection bias and threaten the internal validity of the study. During the revision process, we identified a typographical error in the original manuscript regarding the number of invalid questionnaires (previously stated as "35", which should be corrected to "34"). We sincerely apologize for this oversight and have made the necessary correction.

In direct response to your suggestion, we have conducted a systematic attrition analysis comparing participants who completed both waves of data collection with those who did not (including dropouts and participants providing invalid responses) on all key baseline (T0) variables. These analyses have been added to the "Participants" subsection within the Research Methods section (lines 275–284, page 224).The results demonstrate:

1. No significant differences in Computational Thinking (CT) scores at T0 were observed between completers and non-completers: t (299) = 0.727, p = .468.
2. No significant differences in AI Literacy (AIL) scores at T0 were found between the two groups: t (299) = -0.949, p = .343.
3. A chi-square test of homogeneity indicated no statistically significant difference in gender distribution between completers and non-completers: χ²(1) = 0.856, p = .355.

In summary, the absence of systematic differences in baseline measures and demographic characteristics between completers and non-completers suggests that the attrition in this study is consistent with random missingness. Therefore, it is unlikely to substantially threaten the internal validity of our longitudinal analyses.

We sincerely appreciate your rigorous assessment, which has significantly enhanced the scientific rigor and robustness of our study's conclusions.

The specific revised content is provided below:

To assess whether attrition introduced selective bias, independent samples t-tests and chi-square tests of homogeneity were conducted to compare completers and non-completers on baseline measures. The t-test results showed no statistically significant differences between the two groups in computational thinking (CT) scores (t(299) = 0.727, p = 0.468) or AI literacy (AIL) scores (t(299) = –0.949, p = 0.343) at Time T0. Furthermore, the chi-square test indicated no significant difference in gender distribution between completers and non-completers (χ²(1) = 0.856, p = 0.355). These results suggest that the attrition was random rather than systematic. The missing data mechanism can be considered Missing Completely at Random, thereby posing minimal threat to the internal validity of the study.

# **References**

1.	Guo L. Analysis of the Impact of Generative Artificial Intelligence on Education. Chongqing Higher Education Research. 2025:1-10.

2.	Long D, Magerko B, Acm, editors. What is AI Literacy? Competencies and Design Considerations. CHI Conference on Human Factors in Computing Systems (CHI); 2020 2020Apr 25-30; Electr Network2020.

3.	Wang BC, Rau PLP, Yuan TY. Measuring user competence in using artificial intelligence: validity and reliability of artificial intelligence literacy scale. BEHAVIOUR & INFORMATION TECHNOLOGY. 2023;42(9):1324-37. doi: 10.1080/0144929X.2022.2072768. PubMed PMID: WOS:000793075900001.

4.	Marx V. Quest for AI literacy. Nature Methods. 2024;21(8):1412-5. doi: 10.1038/s41592-024-02369-5. PubMed PMID: WOS:001297657900003.

5.	Sun L, Zhou L. Generative Artificial Intelligence Literacy: Conceptual Evolution, Framework Constructionand Enhancement Paths. Modern Distance Education. 2025:1-18. doi: 10.13927/j.cnki.yuan.20250319.001.

6.	Ng JY, Maduranayagam SG, Suthakar N, Li A, Lokker C, Iorio A, et al. Attitudes and Perceptions of Medical Researchers Towards the Use of Artificial Intelligence Chatbots in the Scientific Process: A Large-Scale, International Cross-Sectional Survey. medRxiv. 2024:2024.02. 27.24303462.

7.	Ng DTK, Su J, Leung JKL, Chu SKW. Artificial intelligence (AI) literacy education in secondary schools: a review. Interactive Learning Environments. 2024;32(10):6204-24. doi: 10.1080/10494820.2023.2255228. PubMed PMID: WOS:001061437100001.

8.	Du H, Sun YC, Jiang HZ, Islam A, Gu XQ. Exploring the effects of AI literacy in teacher learning: an empirical study. HUMANITIES & SOCIAL SCIENCES COMMUNICATIONS. 2024;11(1). doi: 10.1057/s41599-024-03101-6. PubMed PMID: WOS:001225953800003.

9.	Lin JC, Shu QJ, Chen R, Gao CL, Xu KQ, Yin KL, et al. Cultivation and practice of computational thinking in Project-based teaching. International Journal of Technology and Design Education. 2025;35(4):1649-61. doi: 10.1007/s10798-024-09954-x. PubMed PMID: WOS:001378325700001.

10.	Wing JM. Computational thinking. COMMUNICATIONS OF THE ACM. 2006;49(3):33-5. doi: 10.1145/1118178.1118215. PubMed PMID: WOS:000236006000014.

11.	Korkmaz Ö, Çakir R, Özden MY. A validity and reliability study of the computational thinking scales (CTS). Computers in Human Behavior. 2017;72:558-69. doi: 10.1016/j.chb.2017.01.005.

12.	Song YH. The Effects of Self-Directed Learning Ability and Computational Thinking on AI Literacy among College Students: A Case Study of K Theological University. Journal of Christian Education in Korea. 2024;80:245-61. PubMed PMID: KJD:ART003149423.

13.	Celik I. Exploring the Determinants of Artificial Intelligence (AI) Literacy: Digital Divide, Computational Thinking, Cognitive Absorption. TELEMATICS AND INFORMATICS. 2023;83. doi: 10.1016/j.tele.2023.102026. PubMed PMID: WOS:001052604600001.

14.	Kandlhofer M, Steinbauer G, Hirschmugl-Gaisch S, Huber P, Ieee. Artificial Intelligence and Computer Science in Education: From Kindergarten to University. 2016 IEEE FRONTIERS IN EDUCATION CONFERENCE (FIE)2016.

15.	Kong S-C, Man-Yin Cheung W, Zhang G. Evaluation of an artificial intelligence literacy course for university students with diverse study backgrounds. Computers and Education: Artificial Intelligence. 2021;2. doi: 10.1016/j.caeai.2021.100026.

16.	Jiang B, Li Z. Effect of Scratch on computational thinking skills of Chinese primary school students. Journal of Computers in Education. 2021;8(4):505-25.

17.	Master A, Tang D, Forsythe D, Alexander TM, Cheryan S, Meltzoff AN. Gender equity and motivational readiness for computational thinking in early childhood. Early Childhood Research Quarterly. 2023;64:242-54. doi: 10.1016/j.ecresq.2023.03.004.

18.	Lai X, Ye J, Wong GKW. Effectiveness of collaboration in developing computational thinking skills: A systematic review of social cognitive factors. Journal of Computer Assisted Learning. 2023;39(5):1418-35. doi: 10.1111/jcal.12845.

19.	Zhang S, Wong GKW. Understanding individual differences in computational thinking development of primary school students: A three‐wave longitudinal study. Journal of Computer Assisted Learning. 2024. doi: 10.1111/jcal.12940.

20.	Sun L, You X, Zhou D. Evaluation and development of STEAM teachers’ computational thinking skills: Analysis of multiple influential factors. Education and Information Technologies. 2023;28(11):14493-527. doi: 10.1007/s10639-023-11777-7.

21.	Sun C, Yang S, Becker B. Debugging in Computational Thinking: A Meta-analysis on the Effects of Interventions on Debugging Skills. Journal of Educational Computing Research. 2024;62(4):1087-121. doi: 10.1177/07356331241227793.

22.	Cutumisu M, Adams C, Glanfield F, Yuen C, Lu C. Using Structural Equation Modeling to Examine the Relationship Between Preservice Teachers’ Computational Thinking Attitudes and Skills. IEEE Transactions on Education. 2022;65(2):177-83. doi: 10.1109/te.2021.3105938.

23.	Zhao L, Wu X, Luo H. Developing AI Literacy for Primary and Middle School Teachers in China: Based on a Structural Equation Modeling Analysis. Sustainability. 2022;14(21). doi: 10.3390/su142114549.

24.	Velander J, Taiye MA, Otero N, Milrad M. Artificial Intelligence in K-12 Education: eliciting and reflecting on Swedish teachers' understanding of AI and its implications for teaching & learning. Education and Information Technologies. 2023;29(4):4085-105. doi: 10.1007/s10639-023-11990-4.

25.	Apiola M, Sutinen E. Design science research for learning software engineering and computational thinking: Four cases. Computer Applications in Engineering Education. 2021;29(1):83-101. doi: 10.1002/cae.22291. PubMed PMID: WOS:000541227700001.

26.	Romero M. Lifelong learning challenges in the era of artificial intelligence: A computational thinking perspective. arXiv preprint arXiv:240519837. 2024.

27.	Lin X-F, Zhou Y, Shen W, Luo G, Xian X, Pang B. Modeling the structural relationships among Chinese secondary school students' computational thinking efficacy in learning AI, AI literacy, and approaches to learning AI. Education and Information Technologies. 2024;29(5):6189-215. doi: 10.1007/s10639-023-12029-4. PubMed PMID: WOS:001039406700001.

28.	Greenwald E, Leitner M, Wang N, editors. Learning artificial intelligence: Insights into how youth encounter and build understanding of AI concepts. Proceedings of the AAAI Conference on Artificial Intelligence; 2021.

29.	Kim S, Jang Y, Choi S, Kim W, Jung H, Kim S, et al. Analyzing teacher competency with TPACK for K-12 AI education. KI-Künstliche Intelligenz. 2021;35(2):139-51.

30.	Lindell MK, Whitney DJ. Accounting for common method variance in cross-sectional research designs. The Journal of applied psychology. 2001;86(1):114-21. doi: 10.1037/0021-9010.86.1.114. PubMed PMID: MEDLINE:11302223.

31.	Wu K, Vassileva J, Zhao Y. Understanding users' intention to switch personal cloud storage services: Evidence from the Chinese market. Computers in Human Behavior. 2017;68:300-14. doi: 10.1016/j.chb.2016.11.039. PubMed PMID: WOS:000392767400029.

32.	Hsu J-Y, Chen C-C, Ting P-F. Understanding MOOC continuance: An empirical examination of social support theory. Interactive Learning Environments. 2018;26(8):1100-18. doi: 10.1080/10494820.2018.1446990. PubMed PMID: WOS:000447196500009.

33.	Cheng S, Lee S-J, Choi B. An empirical investigation of users' voluntary switching intention for mobile personal cloud storage services based on the push-pull-mooring framework. Computers in Human Behavior. 2019;92:198-215. doi: 10.1016/j.chb.2018.10.035. PubMed PMID: WOS:000457504100020.

34.	Lin C-L, Jin YQ, Zhao Q, Yu S-W, Su Y-S. Factors Influence Students' Switching Behavior to Online Learning under COVID-19 Pandemic: A Push-Pull-Mooring Model Perspective. Asia-Pacific Education Researcher. 2021;30(3):229-45. doi: 10.1007/s40299-021-00570-0. PubMed PMID: WOS:000641225600001.

35.	Wang L, Zhang Q. Investigating the Impact of the Time Interval Selection on Autoregressive Mediation Modeling: Result Interpretations, Effect Reporting, and Temporal Designs. Psychological Methods. 2020;25(3):271-91. doi: 10.1037/met0000235. PubMed PMID: WOS:000537174900002.

36.	Wu X, Li R, Liu K, Luan J. Bridging the Digital Divide: A Longitudinal Empirical Study on Enhancing AI Literacy among Vocational College Students. ournal of Vocational Education. 2025;41(05):87-96.

37.	Zhao HH, Shipp AJ, Carter K, Gonzalez-Mulé E, Xu E. Time and change: A meta-analysis of temporal decisions in longitudinal studies. Journal of Organizational Behavior. 2024;45(4):620-40. doi: <https://doi.org/10.1002/job.2771>.

38.	Laupichler MC, Aster A, Raupach T. Delphi study for the development and preliminary validation of an item set for the assessment of non-experts' AI literacy. Computers and Education: Artificial Intelligence. 2023;4. doi: 10.1016/j.caeai.2023.100126.

39.	Laupichler MC, Aster A, Meyerheim M, Raupach T, Mergen M. Medical students' AI literacy and attitudes towards AI: a cross-sectional two-center study using pre-validated assessment instruments. BMC MEDICAL EDUCATION. 2024;24(1). doi: 10.1186/s12909-024-05400-7. PubMed PMID: WOS:001199941000005.

40.	Ng DTK, Lee M, Tan RJY, Hu X, Downie JS, Chu SKW. A review of AI teaching and learning from 2000 to 2020. Education and Information Technologies. 2023;28(7):8445-501. doi: 10.1007/s10639-022-11491-w. PubMed PMID: WOS:000901853400003.

41.	Xia Q, Chiu TKF, Chai CS, Xie K. The mediating effects of needs satisfaction on the relationships between prior knowledge and self-regulated learning through artificial intelligence chatbot. British Journal of Educational Technology. 2023;54(4):967-86. doi: 10.1111/bjet.13305. PubMed PMID: WOS:000921663100001.

42.	Zhang H, Perry A, Lee I. Developing and Validating the Artificial Intelligence Literacy Concept Inventory: an Instrument to Assess Artificial Intelligence Literacy among Middle School Students. International Journal of Artificial Intelligence in Education. 2025;35(1):398-438. doi: 10.1007/s40593-024-00398-x.

43.	Ng DTK, Su J, Chu SKW. Fostering Secondary School Students' AI Literacy through Making AI-Driven Recycling Bins. Education and Information Technologies. 2024;29(8):9715-46. doi: 10.1007/s10639-023-12183-9. PubMed PMID: WOS:001067366500002.

44.	Kong S-C, Cheung M-YW, Tsang O. Developing an artificial intelligence literacy framework: Evaluation of a literacy course for senior secondary students using a project-based learning approach. Computers and Education: Artificial Intelligence. 2024;6:100214. doi: <https://doi.org/10.1016/j.caeai.2024.100214>.

45.	Chiu TKF, Chen Y, Yau KW, Chai C-s, Meng H, King I, et al. Developing and validating measures for AI literacy tests: From self-reported to objective measures. Computers and Education: Artificial Intelligence. 2024;7:100282. doi: <https://doi.org/10.1016/j.caeai.2024.100282>.

46.	Laupichler MC, Aster A, Schirch J, Raupach T. Artificial intelligence literacy in higher and adult education: A scoping literature review. Computers and Education: Artificial Intelligence. 2022;3:100101. doi: <https://doi.org/10.1016/j.caeai.2022.100101>.

47.	Su J, Yang W. Artificial Intelligence (AI) literacy in early childhood education: an intervention study in Hong Kong. Interactive Learning Environments. 2024;32(9):5494-508. doi: 10.1080/10494820.2023.2217864. PubMed PMID: WOS:000999989100001.

48.	Yim IHY, Su J. Artificial intelligence (AI) learning tools in K-12 education: A scoping review. Journal of Computers in Education. 2025;12(1):93-131. doi: 10.1007/s40692-023-00304-9.
