# Supplementary material for: The impact of university students’ computational thinking on AI literacy: A longitudinal study based on SEM-PLS
Source: PLoS One. 2026 May 27;21(5):e0350124. doi: 10.1371/journal.pone.0350124 (PMC13215501; doi:10.1371/journal.pone.0350124)
Supplement: Attachment — Submitted filename: Response_to_Reviewers_auresp_2.docx [file pone.0350124.s005.docx]

Subject: Rebuttal Letter: Manuscript PONE-D-25-21913R1

Dear Editor and Reviewers,

Thank you for granting us the opportunity to revise our manuscript titled “The Impact of College Students' Computational Thinking on Artificial Intelligence Literacy: A Longitudinal Study Based on SEM-PLS” (Manuscript ID: PONE-D-25-21913R1). We sincerely appreciate the valuable time and effort you and the reviewers have invested in providing insightful and constructive feedback on our work.

We have carefully reviewed all comments and made comprehensive revisions to the manuscript. We believe these modifications have significantly enhanced the quality and clarity of the paper.

Below is our point-by-point response to each comment raised by the reviewers. All modifications in the manuscript are highlighted in red for your reference.

Responses to Reviewer2

(The reviewer's original comments are displayed in blue, while the revised responses are highlighted in red.)

# **Reviewer #2**

## **Comment 1:**

There are quite a few typos.

## **Response 1:**

We appreciate the reviewers' valuable feedback on language expression. Accordingly, we have proofread and polished the entire manuscript to enhance readability by correcting spelling and grammatical errors. We hope the revised version is now clearer and more accessible for readers to understand.

## **Comment 2:**

Also, while you're performing a t-test, this requires normality, so please include the results of normality tests.

## **Response 2:**

We agree with the reviewer that this is an important methodological point. As suggested, we performed the Shapiro-Wilk test to assess the normality of the baseline continuous variables. The results indicated that the distributions for the CT and AIL scores significantly deviated from normality (p < 0.05).

Therefore, to ensure the robustness of our findings, we additionally conducted nonparametric Mann-Whitney U tests for verification. The results from these nonparametric tests were fully consistent with the original t-test findings:

- CT score: U = 8082.500, p = 0.105
- AIL score: U = 8517.500, p = 0.329

We have now integrated both the normality test results and the results of the Mann-Whitney U tests into the revised manuscript. These revisions are located between lines 275 and 278 on page 13.

This addition strengthens our methodological rigor and confirms that our initial conclusions are valid irrespective of the parametric assumptions.

## **Comment 3:**

This is the main revision. In the abstract, it states “novel contribution...establishing a longitudinal link,” but the longitudinal hypothesis H3 (CT at T0 → AIL at T1) is not significant (β=0.021, p=0.708). Therefore, this claim of novelty is inconsistent with the current results. Consequently, the claim does not hold. Please address this inconsistency.

## **Response 3:**

We greatly appreciate the reviewer's critical and insightful comment. The reviewer's perspective is entirely correct. We acknowledge that the original assertion of “establishing a longitudinal association” was overly assertive and not supported by the non-significant results of H3.

To address this issue, we have taken the following actions:

We have revised the statement in the abstract. The original sentence has been modified to: “This study employs a longitudinal research approach to elucidate the complex relationship between computational thinking and AI literacy, providing theoretical insights and methodological guidance for educational practice, thereby contributing to the literature in this field.” These revisions are located between lines 39 and 42 on page 2. The new phrasing more accurately reflects the exploratory longitudinal design of this study and avoids overstating the research findings.

In the discussion section (lines 425–457, pages 20–22), we explicitly explain the reasons for the lack of significance in the longitudinal study (H3 hypothesis), proposing two core interpretations:

Multifactorial nature of learning: We note that in real educational settings, the development of AI literacy is influenced by multiple factors (e.g., curriculum content, peer interactions, teacher guidance), which may have weakened the independent predictive power of baseline computational thinking during the intervention period.

Methodological considerations: We analyzed the potential impact of ceiling effects in short-term longitudinal designs and the sensitivity of AI literacy measurement tools.

We believe these revisions not only resolve inconsistencies in the findings but also enhance the study's academic contribution by deepening our understanding of the relationship between critical thinking and autonomous learning.
