# Supplementary material for: The impact of university students’ computational thinking on AI literacy: A longitudinal study based on SEM-PLS
Source: PLoS One. 2026 May 27;21(5):e0350124. doi: 10.1371/journal.pone.0350124 (PMC13215501; doi:10.1371/journal.pone.0350124)
Supplement: Attachment — Submitted filename: Response_to_Reviewers_auresp_3.docx [file pone.0350124.s006.docx]

Subject: Rebuttal Letter: Manuscript PONE-D-25-21913R2

Dear Dr. Salas-Velasco and Reviewers,

Thank you for giving us the opportunity to revise our manuscript entitled “The Impact of University Students’ Computational Thinking on AI Literacy: A Longitudinal Study Based on SEM-PLS” (Manuscript ID: PONE-D-25-21913R2). We greatly appreciate the time and effort that you and the reviewers have dedicated to providing insightful and constructive comments on our manuscript.

We have carefully considered all the comments and have made extensive revisions to the manuscript accordingly. We believe that these revisions have significantly improved the quality and clarity of our manuscript.

Below, we provide a point-by-point response to all the comments raised by the reviewers. All changes in the manuscript have been highlighted in red for your convenience.

Responses to Editor and Reviewers

(Original comments by reviewers are in blue and modified responses are marked in red.)

# **Editor**

## **Comment 1:**

Light stylistic refinement of the abstract (if desired).

## **Response 1:**

Thank you for this suggestion. We have made an effort to refine the abstract in terms of style and conciseness while trying to preserve the core findings and meaning. The language has been lightly polished with the aim of improving its flow and readability.

## **Comment 2:**

Minor language polishing.

## **Response 2:**

Thank you for this recommendation. We have carefully gone through the manuscript and made minor language revisions throughout the text. We have attempted to improve sentence structure, word choice, and grammatical accuracy to enhance overall clarity.

## **Comment 3:**

Briefly reinforcing the discussion of sample size, timeliness, and broader educational implications of AI, including potential risks, at a conceptual level.

## **Response 3:**

Thank you for this insightful suggestion. In response, we have made two targeted additions to the manuscript.

First, to address sample size and timeliness, we have added the following text in the Limitations section (see page 27, lines 566–584).

Second, to reinforce the broader educational implications and potential risks of AI, we have added the following text in the Discussion section (see page 23, lines 474–489).

We hope these additions help to address the conceptual points raised and contribute to a more balanced discussion.

# **Reviewer #2**

## **Comment 1:**

While not a major issue, please perform careful proofreading once more before final acceptance. For example:

・In Reference 5, the space is missing between “Framework Construction and Enhancement Paths” and Construction and.

・In the conference name of the reference, “WIPSCE 20242024” has duplicate years.

・In the labels of Figure 2 (Structural Model), “T0-Computation thinking”, “T1- Computation thinking” are missing the letter ‘l’ in “Computational”.

## **Response 1:**

We sincerely thank the reviewer for catching these oversights. We have corrected each of the specific errors noted, including the missing space in Reference 5, the repeated year in the conference name, and the spelling of “Computational” in Figure 2. In addition, we have performed another round of careful proofreading across the entire manuscript and made minor language polishing where needed. We hope the manuscript now reads more smoothly and accurately.

## **Comment 2:**

The Shapiro–Wilk test statistic and p-value itself are not reported in the main text. The paragraph above only states “baseline continuous variables might deviate from a normal distribution” without reporting any normality test values.

Furthermore, while the authors emphasize paired t-tests for T0–T1 changes (H4, H5) in this paper, that section (Table 7 and surrounding text) does not include any mention of normality or results from nonparametric tests like the Wilcoxon signed-rank test.

## **Response 2:**

We sincerely thank the reviewer for the rigorous methodological observation. The original manuscript indeed lacked explicit normality reporting and justification for parametric testing. In response, we have made the following revisions:

(1) Attrition analysis: We have removed the independent-samples t-tests and the associated vague statement about possible deviation from normality. Instead, we now rely entirely on nonparametric tests to compare completers and non-completers on baseline measures. Specifically, we report the Mann–Whitney U test results (CT score: U = 8082.500, p = 0.105; AIL score: U = 8517.500, p = 0.329) and the chi-square test of homogeneity for gender distribution (χ²(1) = 0.856, p = 0.355). These results, now transparently reported in the manuscript (see page 13, lines 268–279), support the conclusion that attrition was random rather than systematic.

(2) Paired t-tests: We have added explicit normality evidence for the difference scores used in the T0–T1 comparisons. The Shapiro–Wilk test results (CT: W = 0.988, p = 0.056; AIL: W = 0.996, p = 0.910) indicate that the difference scores do not significantly deviate from normality, thereby supporting the use of parametric paired t-tests. This information is now reported in the relevant section (see page 37, lines 394–396).

We hope these revisions adequately address the reviewer’s concern and improve the overall transparency and rigor of our statistical reporting.

## **Comment 3:**

Regarding the phrasing “positive predictor” in the conclusion, it would be safer to soften it to explicitly indicate a cross-sectional prediction (association at the same time point), such as “cross-sectional models showed that CT is a positive predictor of AIL at each time point.”

## **Response 3:**

We fully agree with this suggestion and thank the reviewer for helping us improve the precision of our wording. We have revised the conclusion accordingly to explicitly anchor the predictive relationship in cross-sectional models, so as not to imply a causal direction. In addition, we have carefully reviewed and made corresponding adjustments to similar phrasing throughout the manuscript to maintain a consistent interpretation. The revised text now appears in the Conclusion section (see page 20, lines 411–414).

# **Reviewer #3**

## **Comment 1:**

Abstract: Please remove numerical values from the abstract, as numbers alone do not communicate the study’s meaning clearly. Instead, the abstract should briefly state the background, purpose, key findings, and especially the implications in a more descriptive way.

## **Response 1:**

Thank you for this suggestion. We have removed all numerical values from the abstract and rewritten it in a more descriptive style. The revised abstract now focuses on conveying the background, purpose, key findings, and implications in textual form (see page 2, lines 29–40).

## **Comment 2:**

Language quality: There are indications that AI tools may have been used in drafting the manuscript. The paper should undergo careful human proofreading (or professional language editing) to improve clarity, coherence, and academic tone before acceptance.

## **Response 2:**

We appreciate this observation and take the concern seriously. We have made efforts to carefully proofread and polish the manuscript throughout to improve clarity, coherence, and academic tone.

## **Comment 3:**

Timeliness of the study: The study appears to be based on data collected in 2024. Given that it is now 2026 and the AI field evolves rapidly, the authors should justify the continued relevance of the findings and/or explicitly acknowledge this as a limitation.

## **Response 3:**

Thank you for this suggestion. We have added a paragraph in the Limitations section that acknowledges the 2024 data collection, notes the rapid evolution of AI, while arguing for the stability of the core cognitive relationship, and advises readers to interpret the findings with the evolving context in mind (see page 28, lines 576–584).

## **Comment 4:**

Sample size and potential bias: The sample size is relatively small. Please explain how the authors ensured the findings are not strongly affected by sampling bias, and discuss how the sample size may influence generalizability. If applicable, add a limitation and suggestions for future research with larger samples.

## **Response 4:**

We appreciate this comment. A discussion has been added in the Limitations section that acknowledges the relatively small sample, explains the sampling procedures employed, outlines the limitations in generalizability, and offers suggestions for future research with larger, more diverse samples (see page 27, lines 566–575).

## **Comment 5:**

Discussion (negative effects of AI): The discussion should also acknowledge and analyze potential negative effects or risks of AI in education (e.g., overreliance, AI dependency, bias, reduced critical thinking, ethical concerns). The discussion can be strengthened by engaging with recent related literature, such as:

(2024). Examining Chinese preservice mathematics teachers’ adoption of AI chatbots for learning: Unpacking perspectives through the UTAUT2 model. Education and Information Technologies.

(2024). Latent profile analysis of AI literacy and trust in mathematics teachers and their relations with AI dependency and 21st-century skills. Behavioral Sciences (Basel, Switzerland), 14.

(2025). Exploring the relationship between AI literacy, AI trust, AI dependency, and 21st-century skills in preservice mathematics teachers. Scientific Reports, 15(1).

## **Response 5:**

Thank you for raising this point. We have added a paragraph in the Discussion section that addresses the potential negative effects and risks of AI in education, including overreliance, dependency, bias, and ethical concerns (see page 23, lines 474–489).
